# Shaping of a three-dimensional carnivorous trap through modulation of a planar growth mechanism

**DOI:** 10.1371/journal.pbio.3000427

**Published:** 2019-10-10

**Authors:** Karen J. I. Lee, Claire Bushell, Yohei Koide, John A. Fozard, Chunlan Piao, Man Yu, Jacob Newman, Christopher Whitewoods, Jerome Avondo, Richard Kennaway, Athanasius F. M. Marée, Minlong Cui, Enrico Coen

**Affiliations:** 1 Department of Cell and Developmental Biology, John Innes Centre, Norwich Research Park, Norwich, United Kingdom; 2 College of Agriculture and Food Science, Zhejiang Agriculture and Forestry University, Linan, Zhejiang, China; 3 Department of Computational and Systems Biology, John Innes Centre, Norwich Research Park, Norwich, United Kingdom; UCSD, UNITED STATES

## Abstract

Leaves display a remarkable range of forms, from flat sheets with simple outlines to cup-shaped traps. Although much progress has been made in understanding the mechanisms of planar leaf development, it is unclear whether similar or distinctive mechanisms underlie shape transformations during development of more complex curved forms. Here, we use 3D imaging and cellular and clonal analysis, combined with computational modelling, to analyse the development of cup-shaped traps of the carnivorous plant *Utricularia gibba*. We show that the transformation from a near-spherical form at early developmental stages to an oblate spheroid with a straightened ventral midline in the mature form can be accounted for by spatial variations in rates and orientations of growth. Different hypotheses regarding spatiotemporal control predict distinct patterns of cell shape and size, which were tested experimentally by quantifying cellular and clonal anisotropy. We propose that orientations of growth are specified by a proximodistal polarity field, similar to that hypothesised to account for *Arabidopsis* leaf development, except that in *Utricularia*, the field propagates through a highly curved tissue sheet. Independent evidence for the polarity field is provided by the orientation of glandular hairs on the inner surface of the trap. Taken together, our results show that morphogenesis of complex 3D leaf shapes can be accounted for by similar mechanisms to those for planar leaves, suggesting that simple modulations of a common growth framework underlie the shaping of a diverse range of morphologies.

## Abstract 2

Many plant and animal organs derive from tissue sheets, but how are they shaped to create the diversity of forms observed in nature? This study uses a combination of imaging and mathematical modelling to show how carnivorous plant traps shape themselves in 3D by a growth framework oriented by tissue polarity, similar to that found in planar leaves.

## Introduction

Many plant and animal organs, such as leaves, flowers, hearts, and wings, derive from tissue sheets. A general question in developmental and evolutionary biology is how tissue sheets are shaped to create such a diversity of forms. A good illustration is leaf development. Leaves exhibit remarkable variation in shape, from simple or compound planar forms to convoluted three-dimensional forms such as those of pitcher plants. The molecular genetic control of leaf shape has been extensively studied for planar forms, with key genes modifying leaf shape identified [1–6]. Variation in patterns of gene activity has also been shown to underlie variation in leaf shape between species [7–10]. Clonal analysis and tracking and monitoring cell division have further revealed spatiotemporal variation in patterns of division and growth, and led to the formulation of models for how shape arises through local variations in rates and orientations of growth [11,12]. However, it is unclear how these models for planar leaf development are related to morphogenetic changes in highly curved 3D leaf forms, such as epiascidiate (cup or tubular-shaped) leaves.

Epiascidiate leaves have evolved four times independently: in the families Nepenthaceae, Sarraceniaceae, Cephalotaceae, and Lentibulariaceae [13,14]. In all these cases, the epiascidiate form is associated with nutrient acquisition from animals (carnivory). Based on comparative anatomy, the inner surface of the epiascidiate leaf is believed to be equivalent to the adaxial surface of a planar leaf, whereas the outer surface is equivalent to the abaxial surface [4,13,15,16]. The petiole of the epiascidiate leaf inserts on the abaxial side, similar to the situation for a peltate leaf [13]. However, the mechanism by which the epiascidiate leaf is initially formed and then shaped during development is poorly understood. Here, we address the developmental mechanisms controlling the second aspect, involving shaping of a highly curved sheet.

In the genus *Utricularia* (Lentibulariaceae), epiascidiate leaves, termed traps, use suction to catch prey, requiring highly coordinated morphogenesis to ensure that the opening and closing mechanisms operate effectively. *Utricularia* has several advantages for analysis of epiascidiate leaf development [17]. The traps are transparent and only a few millimetres long, making them convenient for imaging. Much of the trap comprises only two cell layers [18,19], compared to approximately seven cells for *Arabidopsis* leaves, simplifying growth analysis. The genome of *Utricularia gibba* is among the smallest in plants (100 Mb) and has been fully sequenced, providing a resource for molecular genetic and evolutionary studies [20–27]. *Utricularia* is also a large genus, comprising about 235 species with varying trap shapes, allowing for comparative analysis [28–31].

Snapshots, scanning electron micrographs, and drawings of *Utricularia* traps at various developmental phases have been described [18,32–35]. However, quantitative growth and cellular analysis of morphogenesis have not been carried out. Such studies require the development of transformation methods for introducing fluorescent proteins to mark cell membranes or clones in *Utricularia*, followed by 3D imaging at different developmental stages. Moreover, models need to be developed for how changes in three-dimensional shape and curvature arise, and predictions of these models need to be tested against experimental data.

Here, we develop and apply these approaches to analyse trap morphogenesis in *U*. *gibba*. We show that after forming a near-spherical shape, *U*. *gibba* traps undergo defined changes in shape and curvature. By measuring 3D snapshots of traps at various developmental stages and exploring computational growth models, we show that differential rates and orientations of growth are both likely involved in the observed shape transformations. This hypothesis is further tested by marking cells with green fluorescent protein (GFP) and testing the resulting cell and clone shapes against model predictions. To account for oriented growth, the computational model invokes a proximodistal polarity field that is comparable to that proposed to account for *Arabidopsis* leaf development, except that it propagates within a curved sheet. The proposed polarity field is supported through analysis of quadrifid gland orientations. Our findings thus suggest that simple modulation of mechanisms underlying planar leaf development can account for shaping of more complex 3D leaf shapes, providing a unified explanation for diverse leaf forms.

## Results

### Shape change during trap growth

**Fig 1:**
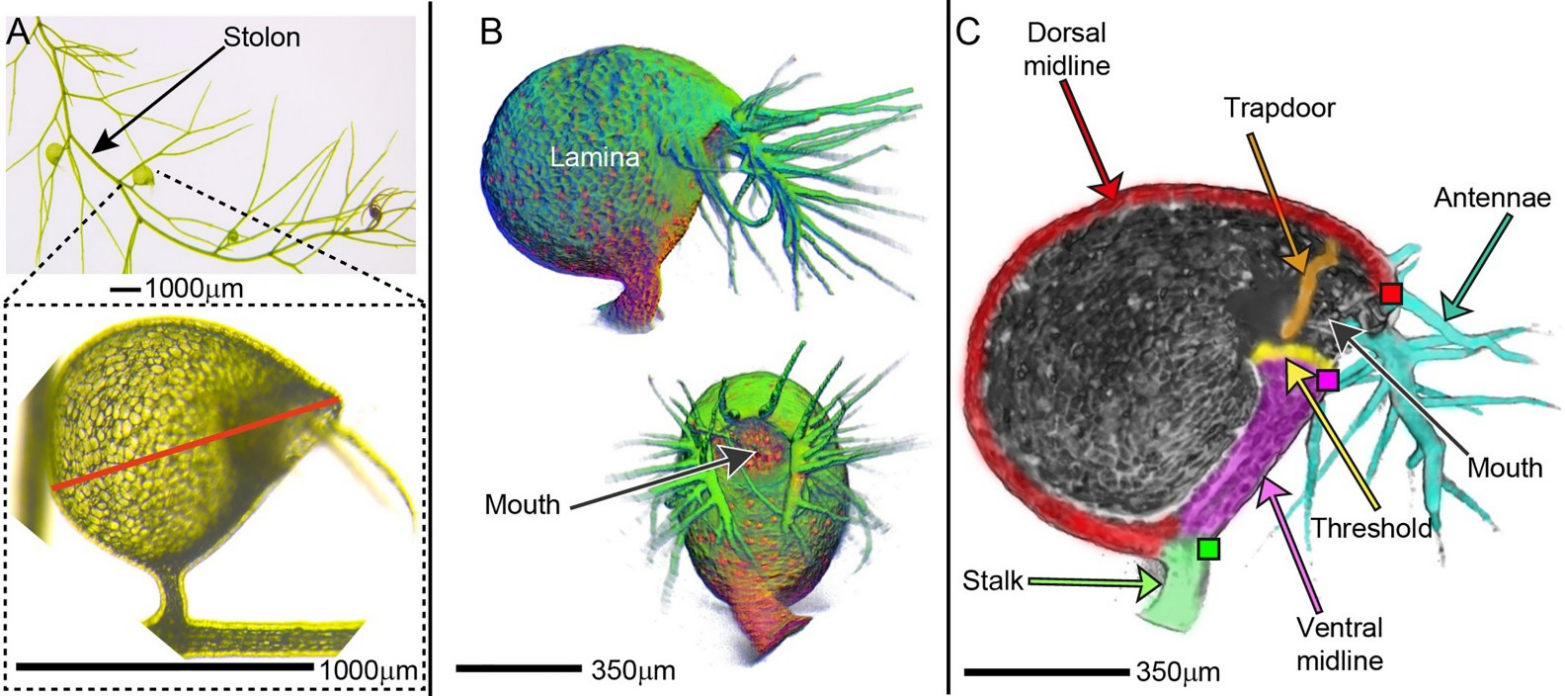
Mature *U*. *gibba* trap. (A) Stolon bearing traps. Insert shows mature trap. Red line drawn from mouth (right) to the furthest point at the back of the trap. (B) OPT volume views of a trap in lateral view (top) or ventral view (bottom). (C) Volume view clipped in the sagittal plane. Coloured squares indicate landmarks: dorsal lip (red), ventral lip (magenta), and stalk indentation (green). Domains between these landmarks are colour-coded as red (dorsal midline), magenta (ventral midline), and green (stalk). Data **https://doi.org/10.6084/m9.figshare.8966153.v1**, Fig 1.7z archive. OPT, Optical Projection Tomography.

*U*. *gibba* traps arise laterally from stolons (Fig 1A) and have a single plane of mirror symmetry [13,29]. Each trap encloses a lumen with an opening at one end, termed the trap entrance or mouth (Fig 1B and 1C). In natural conditions, the lumen of a mature trap is under low internal pressure (primed state) [36,37]. Triggering of the trap door then leads to water intake, together with prey, and equilibration of pressure (relaxed state).

**Fig 2:**
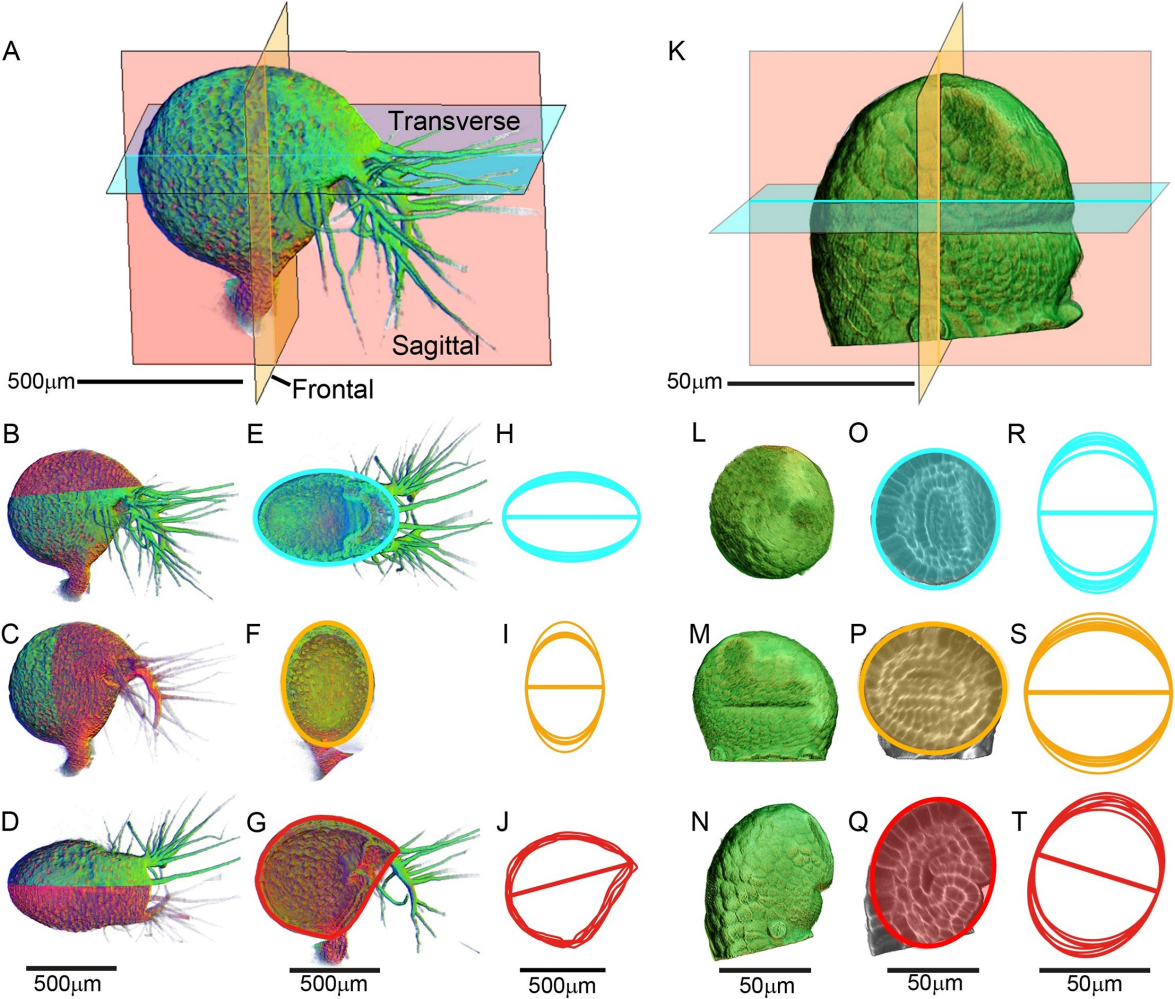
Trap shape at mature and early stages. (A) Volume view of a PI-stained mature *U*. *gibba* trap visualised by OPT. Three section planes are shown. PI fluorescence is red, and tissue autofluorescence is green (S1 Movie). (B–D) Green OPT channel clipped along the transverse (B), frontal (C), and sagittal (D) planes. Red channel is left in place to show region of the trap clipped away in E–G. (E–G) Shapes fitted to circumference in each plane: transverse, cyan (E); frontal, orange (F); sagittal, red (G). (H–J) Superimposed circumferences of six mature traps, colour-coded as in E–G, S1 Data. Ellipses were fitted to transverse and frontal circumferences (H, I). Sagittal circumference was drawn (J). (K) Volume view of young PI-stained *U*. *gibba* trap visualised by confocal microscopy. Three section planes colour-coded as in A, S2 Movie. (L–N) Dorsal (top) (L), ventral (front) (M), and lateral (side) (N) volume views. (O–Q) Ellipses fitted to circumference in each clipped plane colour-coded as in E–G. (R–T) Superimposed circumferences of seven young traps, colour-coded as in E–G, S1 Data. For H–J and R–T, circumferences were manually scaled and rotated to align with a common axis (shown as a line through the middle of each circumference). For both OPT and confocal data, lines were measured in the transverse plane from centre of mouth to back of trap, frontal plane at narrowest region between walls, sagittal plane from dorsal lip to back of trap, S2 Data. Scale bar refers to mean length of the common line. Data **https://doi.org/10.6084/m9.figshare.8966153.v1**, Fig 2.7z archive. OPT, Optical Projection Tomography; PI, propidium iodide.

**Fig 3:**
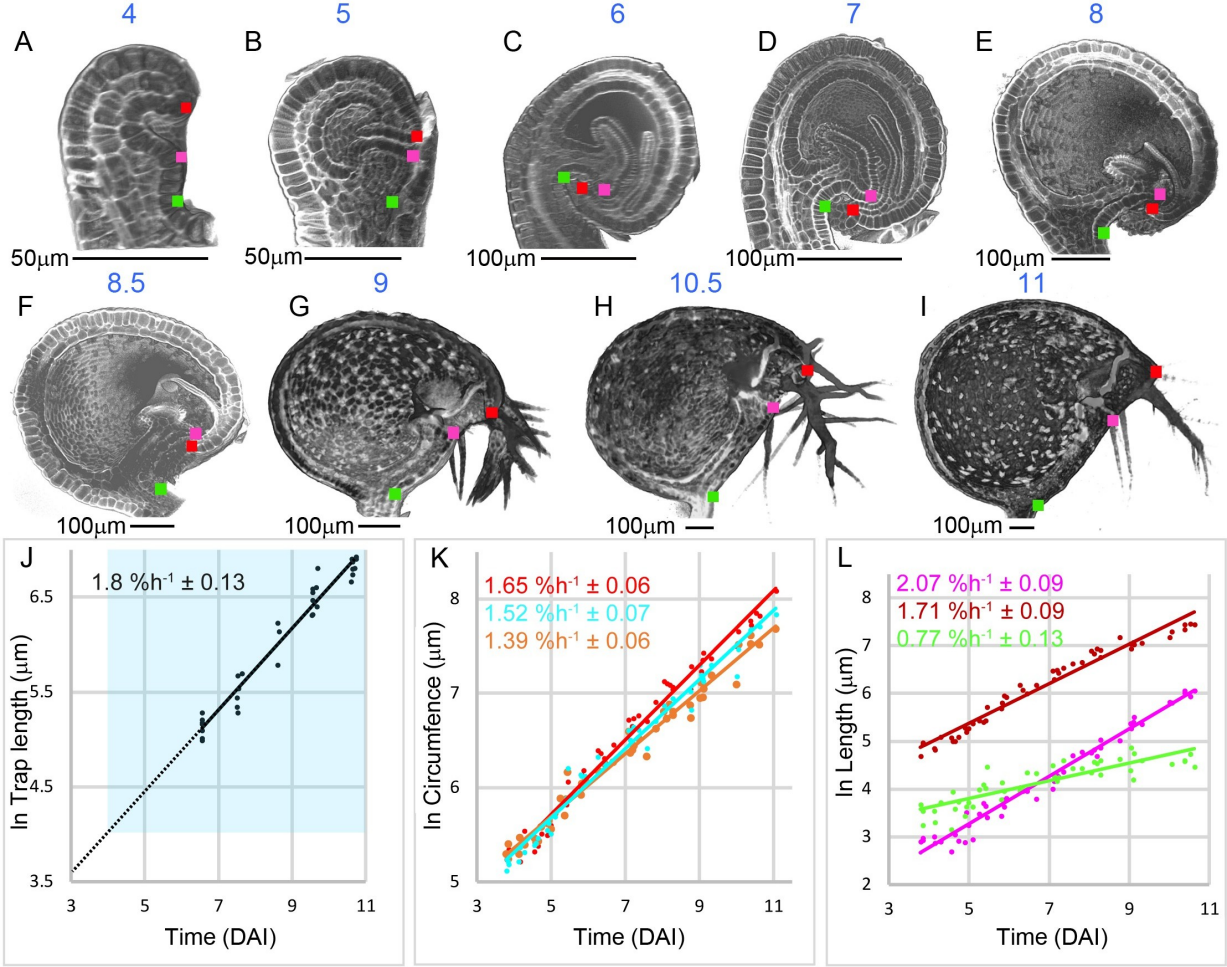
Developmental stages and growth rates. (A–I) Clipped sagittal volume views of traps 4–11 DAI (in blue numbers above trap). Traps were stained with PI and visualised in three dimensions from confocal image stacks (A–F) and OPT reconstructions (G–I). Smaller traps within the circinate apex and traps with occluding stolon tissue were virtually dissected with VolViewer. Coloured squares indicate landmarks as described in Fig 1C. (J–K) Trap growth charts (S3 Data). (J) Natural log of trap length plotted against time for live imaging of traps at daily intervals. A best-fit line was extrapolated back to when the bladder was 10 μm long (dashed line), corresponding to 1–2 cells, which we took to be the initiation stage of the bladder. Mean growth rate was 1.8% h^−1^ ± 0.13 (R^2^ = 0.9607). Blue region shows developmental range of fixed traps analysed in (A–I) and (K, L). (K) Natural log of circumferences measured in VolViewer for transverse (cyan), frontal (orange), and sagittal (red) sections plotted against time (DAI, based on J). Growth rates: 1.52% h^−1^ ± 0.07 (R^2^ = 0.9757, *n* = 46), 1.39% h^−1^ ± 0.06 (R^2^ = 0.9772, *n* = 50), and 1 .65% h^−1^ ± 0.06 (R^2^ = 0.9832, *n* = 52), respectively. For mature traps, where it was not possible to image the entire depth of the trap by confocal microscopy, half the circumference was measured, and this value was doubled to obtain the total circumference. (L) Natural log lengths in sagittal sections for dorsal midline (brick red), ventral midline (magenta), and stalk (green) regions measured in VolViewer and plotted against time (DAI). Growth rates: 2.07% h^−1^ ± 0.09 (R^2^ = 0.9764, n = 51), 1.71% h^−1^ ± 0.09 (R^2^ = 0.9665, *n* = 51), and 0.77% h^−1^ ± 0.13 (R^2^ = 0.7527, *n* = 49). Mature traps showed 5.78% ± 0.45 shrinkage when prepared for OPT (S9 Data). To compensate for this shrinkage, trap-length measurements of all fixed traps were increased by 5.78% before calculating DAI. Data: **https://doi.org/10.6084/m9.figshare.8966153.v1**, Fig 3.7z archive. DAI, days after initiation; OPT, Optical Projection Tomography.

To define the shape and structure of the mature trap, we imaged it in three dimensions using Optical Projection Tomography (OPT) [38]. Under the conditions used for imaging, the trap was in a relaxed state (S1 Fig). In accordance with previous nomenclature [13], the trap could be subdivided into several domains: ventral midline, dorsal midline, lamina, stalk, threshold, and trap door (Fig 1B and Fig 1C). The mouth was decorated with multicellular appendages (antennae). To assess overall trap shape, OPT images were sectioned in three planes: transverse, frontal, and sagittal (Fig 2A, S1 Movie). In transverse section, the mature trap circumference was approximately elliptical (Fig 2B and 2E), confirmed by superimposing circumferences from five traps (Fig 2H, S1 Data, S2 Data). Similarly, the mature trap circumference was elliptical in frontal sections (Fig 2C and 2F, Fig 2I, S1 Data, S2 Data). In sagittal sections, the circumference had a straight edge, corresponding to the ventral midline and mouth (Fig 2D and 2G, and Fig 2J). We compared these circumferences to those from traps at an early developmental stage (Fig 2K, S2 Movie). At this stage, the circumference was approximately circular in all sections, indicating a near-spherical shape (Fig 2L–2T). Intermediate stages of development are illustrated in Fig 3A–3I. Thus, as a first approximation, trap morphogenesis during these stages involves transformation of a near-spherical overall shape to an oblate spheroid (a sphere that has been squashed along one axis) with a straightened edge.

### Growth rates during trap development

To explore hypotheses for how transformations in trap shape arise, we used a computational modelling approach. Such an approach is more powerful if constrained by known growth rate measurements. To obtain these measurements, we first established a temporal framework for trap development by following the growth of individual traps.

At very early phases of development, traps were hidden from view because they were held within a spiral structure, termed the circinate apex [39]. We imaged traps at daily intervals from when they emerged from the spiral until they reached maturity. Trap length was estimated according to the distance from the dorsal lip (Fig 1C, red square) to the furthest point at the back of the trap (Fig 1A, red line).

Plotting log of trap length against time gave an estimated strain rate (relative growth rate) of 1.8% h^−1^ ± 0.13 (Fig 3J, S3 Data, all measured rate estimates are given with ± twice the standard deviation). The growth curve was extrapolated back in time (Fig 3J, dotted line) to define an initiation time (0 days after initiation [DAI]), corresponding to a length of 10 μm (i.e., approximately 1–2 cells). Using this growth curve, a standard time in DAI could be assigned to any trap based on its length (blue region, Fig 3J, S4 Data).

The above framework allowed us to determine strain rates for various trap domains that could later be used to constrain parameters in growth models. We first measured circumferences in the three section planes at different stages of development (Fig 3K, S3 Data). The strain rate was higher along the sagittal circumference (1 .65% h^−1^ ± 0.06) compared to the other circumferences (1.52% h^−1^ ± 0.07 and 1.39% h^−1^ ± 0.06). The sagittal section was further divided into three subdomains based on three landmarks that could be identified throughout development (Fig 1C and Fig 3A–3I, green, magenta, and red squares). These landmarks allowed three domains to be defined: ventral midline, dorsal midline, and stalk diameter (Fig 1C, magenta, red, and green domains). Strain rates for these regions were then estimated from the staged traps (Fig 3L, S3 Data). The ventral midline grew faster (2.07% h^−1^ ± 0.09) than the dorsal midline (1.71% h^−1^ ± 0.09), and the stalk diameter grew the slowest (0.77% h^−1^ ± 0.13).

### Tissue-level modelling

To explore hypotheses that might underlie the observed morphogenetic changes, we developed a series of models constrained by the experimental growth rate data. Models were kept as simple as possible, with hypothetical factors (for example, Midline factor [MID], Stalk factor [STK], Ventral factor [VEN]) being successively introduced to give a clear indication of what contributes to the resulting shape changes. Although model parameters were constrained by experimental data, changes in tissue curvature generated by the model were not specified but were an emergent property arising through mechanical constraints of tissue connectivity.

We used the growing polarised tissue (GPT) modelling framework, in which tissue is treated as a continuous sheet of material with defined thickness, termed the canvas [40]. It is assumed that for each region of the tissue, there is a specified rate of growth that defines how much that region would grow in mechanical isolation from neighbouring tissue. This rate of specified growth is a tensor quantity representing the possibility that the growth (strain rate) may be by different amounts in different directions. Resultant growth is how each region grows in the context of mechanical constraints arising from connectivity with other regions and includes anisotropies, rotations, and curvature that emerge from such constraints [41]. Specified growth, therefore, refers to the intrinsic or active properties of a region, which may be influenced by local gene expression, while resultant growth also includes the passive changes that arise through mechanical connectivity with other regions. Either type of growth can be isotropic (equal in all directions) or anisotropic (greater in some orientations than others). Regional factors in the canvas can modulate specified growth rates, allowing various patterns of growth to be established.

Computationally, the problem is to calculate the deformation field or resultant growth (i.e., a mapping of each region of the tissue to its new position) that will result from applying the field of specified growth rate for all regions when mechanically connected together over some small time interval. In general, there will be no deformation field in which every region of the tissue achieves its specified growth. The difference between the specified and resultant growth is the residual strain and produces a proportionate residual stress. The actual deformation resulting from the field of specified growth is taken to be whatever shape minimizes the residual strain energy. Residual strain is assumed to dissipate after each growth step, reflecting the irreversible plastic flow involved in plant growth [42].

Changes in curvature can arise if regions of the tissue are specified to grow at different rates and/or directions. For example, if a region with high specified areal growth rate is surrounded by regions with low specified growth rate, the tissue may buckle, with the faster-growing region bulging out. More recently, another type of buckling has been described in which specified areal growth rates can be uniform but in which directions of specified anisotropic growth vary [43]. To distinguish between these two causes of buckling, the terms areal and directional conflict resolution have been proposed [44]. In both cases, changes in curvature arise through differential growth properties between regions, with conflicting growth patterns preventing each region from attaining its specified growth rate. Buckling or curvature helps reduce this conflict (reduces strain energy) by allowing each region to grow more closely to its specified rate. Areal conflicts arise when there are nonuniform specified areal growth rates (which may be isotropic or anisotropic), whereas directional conflict can arise when there are nonuniform orientations of growth (even if areal growth rates are uniform). Directional conflicts, therefore, necessarily require specified anisotropy, whereas areal conflicts do not. We explore the ability of each type of tissue conflict resolution, areal or directional, to account for the observed growth rates and shape changes of the *U*. *gibba* trap.

**Fig 4:**
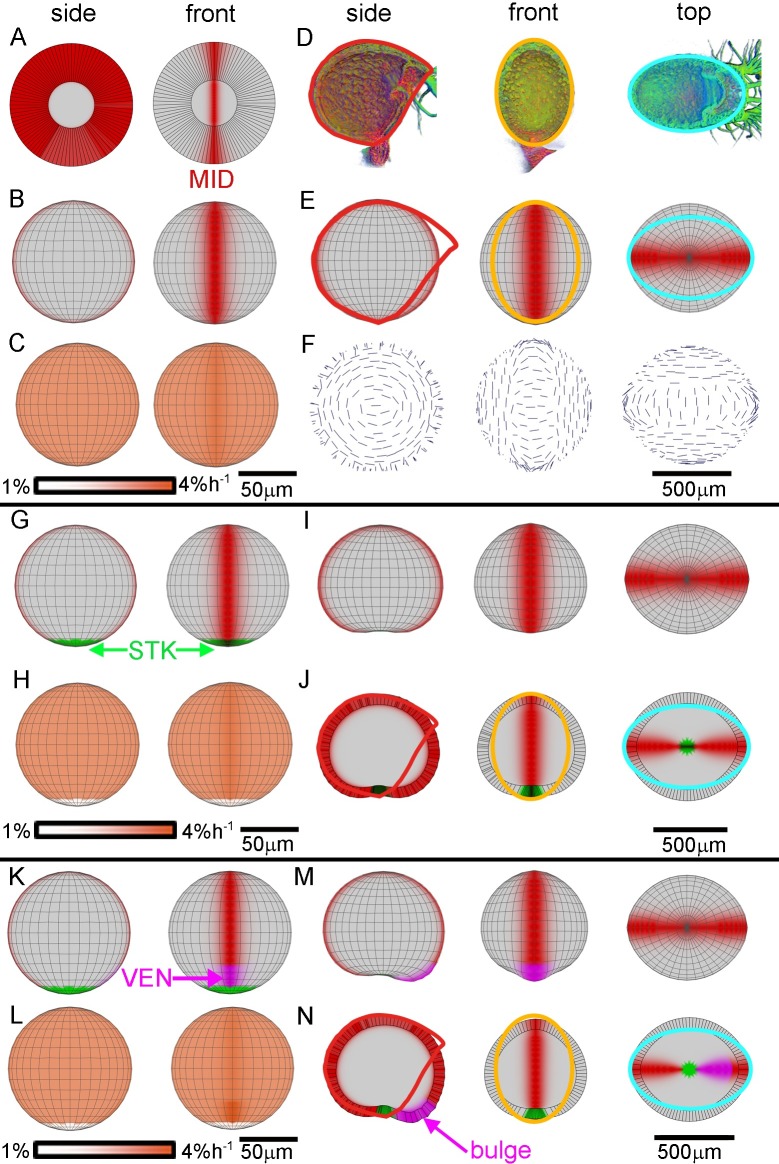
Tissue-level modelling of trap development through areal conflict resolution. Specified growth is isotropic in all cases. Initial spherical canvas is shown from side and front in the left two columns. Resultant shapes from side, front, and top are shown in right three columns, with experimentally observed circumferences (red, orange, cyan) superimposed in E, J, and N to allow comparison between model and data. (A–F) Growth promoted along sagittal circumference by MID, yielding oblate spheroid. (A) Initial canvas sphere clipped to show MID domain (red). Canvas wall thickness = 30 μm. (B) Initial canvas unclipped, showing MID domain. (C) Initial canvas showing specified areal growth rate promoted by MID (note darker orange in MID region). (D) Mature trap clipped views shown in Fig 2E–2G, with shapes fitted to circumference in each plane: sagittal, red; frontal, orange; and transverse, cyan. (E) Resultant canvas shape (oblate spheroid) showing MID domain, with mature trap shape outlines fitted. (F) Resultant canvas shape with major orientations of resultant growth shown as lines. Lines are oriented perpendicular to the MID (sagittal) circumference. (G–J) Growth promoted by MID and inhibited by STK. (G) Initial canvas showing STK domain (green) as well as MID. (H) Initial canvas showing specified areal growth rate promoted by MID and inhibited by STK (note white area at the ‘South Pole’). (I) Resultant canvas shape showing indentation in STK region. (J) Midsection clipped views of into resultant shape. (K–N) Growth promoted by MID and VEN and inhibited by STK. (K) Initial canvas showing VEN domain (magenta) as well as MID and STK. (L) Initial canvas showing specified areal growth rate promoted by MID and VEN and inhibited by STK (note darker orange in VEN domain). (M) Resultant canvas shape showing ventral bulge. (N) Midsections of resultant canvas, with bulge highlighted in the side view (sagittal section). Models: http://cmpdartsvr3.cmp.uea.ac.uk/wiki/BanghamLab/index.php/Software and **https://doi.org/10.6084/m9.figshare.8966153.v1**, Models.7z archive. MID, Midline factor; STK, Stalk factor; VEN, Ventral factor.

In all models that follow, the initial canvas was a hollow sphere with a uniform wall thickness of 30 μm and a diameter of 100 μm, corresponding to the approximate trap shape at 4 DAI (Fig 2K–2T and Fig 4A). To assist with visualisation in three dimensions, a grid of latitudes and longitudes was superimposed on the initial canvas. All regional factors, as well as the gridlines, were fixed to the canvas and deformed with it during growth.

### Shape transformation through areal conflict resolution

We first explored models in which specified growth rates in the plane of the canvas are equal in all directions (isotropic) but can differ between regions, creating potential areal conflicts. Starting from an initial near-spherical shape, one of the key changes during trap morphogenesis is formation of elliptical circumferences in the transverse and frontal planes (Fig 2H and 2I). To a first approximation, this represents a transformation from a sphere to an oblate spheroid. In an oblate spheroid, the two elliptical circumferences are shorter than the circular circumference. In principle, transformation from sphere to oblate spheroid could arise if specified areal growth rate is faster along one circumference, causing the sphere to flatten. This faster-growing circumference could correspond to the sagittal circumference of the *Utricularia* trap because this ends up being longer than the other circumferences and grows at a faster rate (Fig 3K, S3 Data).

To explore this idea, we introduced a factor, MID, expressed along the sagittal circumference or midline of the canvas (Fig 4A and 4B, red). The concentration of MID was set to be highest at the midline and gradually declined away from it. We next needed to constrain parameters in the model according to observed circumferential growth rates. The areal strain rate for a region of planar tissue is the sum of the two linear strain rates in orthogonal directions within the plane. We therefore set the basic specified areal strain rate of our canvas to the sum of the transverse and frontal circumferential strain rates (2.9% h^−1^) according to parameter *b*_*planar*_ (Fig 4C; see model description in Materials and Methods for full details). This strain rate was promoted by MID to give a value of twice the sagittal circumferential strain rate (3.3% h^−1^) along the midline, according to parameter *p*_*mid*_. Specified growth rate in thickness was assumed to be uniform throughout the canvas and set to an experimentally determined average of 0.5% h^−1^, according to parameter *b*_*thickness*_ (S2 Fig, S10 Data). Thus, the model had a total of three parameters (*b*_*planar*_, *p*_*mid*_, *b*_*thickness*_) controlling specified growth rates, each of which was experimentally constrained. These constraints were sufficient for the model because specified growth rate was assumed to be uniformly affected by a given factor.

Running this model led to a transformation of the initial sphere to an oblate spheroid (Fig 4E, S3 Movie). The shape broadly matched that of the front and top views of the mature trap but lacked the straight ventral edge seen in side view (Fig 4D, compare to sagittal shape outline in red). The resultant areal strain rate in the central MID region was slightly lower than that specified (3.18% h^−1^ compared to 3.3% h^−1^). This deficit arises because the slower growth of the rest of the canvas constrained growth of the MID region, even with the change in curvature (i.e., the areal conflict was not fully resolved). For this reason, in subsequent models, we adjusted parameter values for specified growth rates by trial and error in such a way that they gave resultant strain rates that matched experimental strain rate measurements when the model was run. Unresolved areal conflict also introduced slight resultant anisotropy in the pattern of growth, indicated by the field of maximal growth orientations (Fig 4F). These differences between specified and resultant growth highlight emergent features arising through mechanical constraints.

In the above model, the region of the midline that intersected with the stalk was specified to grow at the same rate as the rest of the midline. However, the strain rate for stalk diameter was measured to be lower than other midline regions (0.77% h^−1^; Fig 3L, S3 Data). We therefore introduced an additional factor, STK, at the ‘South Pole’ of the canvas (Fig 4G, green), which inhibited specified areal strain rate according to parameter *h*_*stk*_ (Fig 4H). The result of running this four-parameter model (*b*_*planar*_, *p*_*mid*_, *b*_*thickness*_, *h*_*stk*_) was an oblate spheroid with a slight inflexion at the STK domain caused by the areal conflict between the slower-growing STK domain and its surroundings (Fig 4I and 4J; compare to mature trap outlines shown in red, orange, and cyan and S4 Movie).

**Fig 5:**
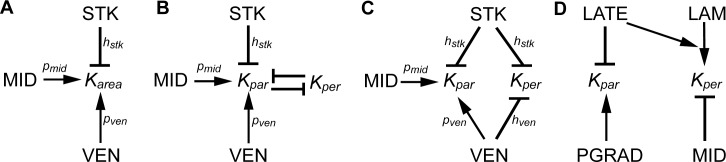
KRNs for tissue-level models. All models have two basic parameters: *b*_*planar*_ = basic areal growth rate and *b*_*thickness*_ = basic growth rate in thickness. The further parameters specific for each model are shown in the individual KRNs. Arrows indicate promotive effects, and blunt ends indicate inhibitory effects. (A) KRN for areal conflict model, with MID and VEN promoting specified areal growth rate and STK inhibiting specified areal growth rate. (B) KRN for directional conflict model, with MID and VEN promoting specified growth rate parallel to the polarity and STK inhibiting specified growth rate parallel to the polarity. Note that to maintain constant specified areal growth rate, an increase in *K*_*par*_ has to be compensated for by a corresponding decrease in *K*_*per*_ (indicated by mutual inhibition). (C) Integrated model. Regulation of *K*_*par*_ and *K*_*per*_ is separable. MID promotes *K*_*par*_. STK inhibits both *K*_*par*_ and *K*_*per*_, and VEN promotes *K*_*par*_ and inhibits *K*_*per*_. There is also a further parameter that influences the width of the VEN domain (*t*_*ven*_) (not shown). (D) KRN for *Arabidopsis* leaf model for comparison. The MID factor for the *Arabidopsis* model is expressed in the midline region and has a higher level of expression in the proximal half of the primordium. The LAM factor is expressed in the presumptive lamina, which occupies most of the primordium except for its most proximal region. PGRAD has a graded distribution that decreases from proximal to distal positions. LATE is expressed uniformly and increases with time.

In the output of the above model, the mouth region remained close to the stalk, in contrast to the observed displacement of the mouth at later stages (Figs 2G and 3I). This displacement of the mouth reflects the higher strain rate of the ventral midline of 2% h^−1^ (Fig 3L, S3 Data.). To account for these observations, we introduced a ventral midline factor, VEN (Fig 4K, magenta), which promoted specified areal strain rate according to parameter *p*_*ven*_ (Fig 4L), giving a resultant strain rate along the ventral midline of approximately 2% h^−1^. The result of running this five-parameter model (interactions summarised in Fig 5A) is a trap with a longer ventral midline that bulges out (Fig 4M and 4N; compare to mature trap sagittal shape outline [red] and S5 Movie), unlike the straightened ventral midline of the real trap (Figs 4D and 1). Bulging arises through the areal conflict caused by the ventral midline region growing faster in all directions than its surroundings. Thus, an areal conflict model can account for the overall flattening of the sphere to create an oblate spheroid but does not readily account for shape of the ventral midline.

### Shape transformation through directional conflict resolution

To determine whether a model based purely on directional conflict resolution might better account for the observed morphogenetic changes, we kept specified areal growth rates uniform (3% h^−1^, defined by a slightly modified value of *b*_*planar*_) and varied specified rates of growth in different orientations (specified anisotropy). Although specified areal growth rates were uniform, resultant areal growth rates need not have been because directional conflicts can lead to some regions growing faster or slower than the rate specified. Specified growth rate in thickness (*b*_*thickness*_) was the same as for the areal conflict model.

**Fig 6:**
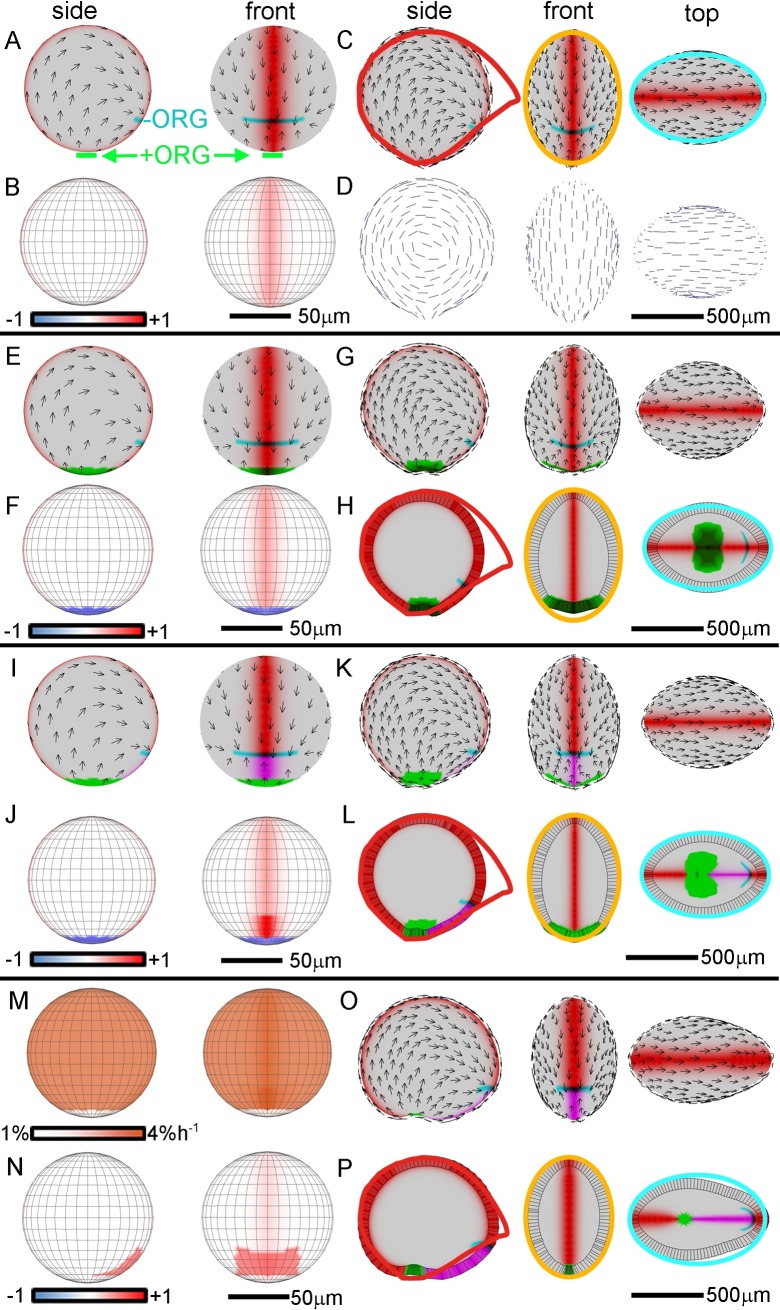
Tissue-level modelling of trap development through directional conflict resolution. Specified areal growth rate is uniform in A–L (directional conflict model) but not in M–P (integrated model). Initial spherical canvas is shown from side and front in the left two columns. Resultant shapes from side, front, and top are shown in right three columns, with experimentally observed circumferences (red, orange, cyan as shown in Fig 4D) superimposed in C, H, L, and P to allow comparison between model and data. (A–D) Directional conflict resolution with anisotropy promoted by MID generating oblate spheroid. (A) Initial canvas showing MID domain (red), polarity (black arrows), and +/−ORGs. Polarity flows from +ORG (green) at the ‘South Pole’ towards −ORG (cyan) at the mouth. (B) Initial canvas showing specified anisotropy, defined as (*K*_*par*_ − *K*_*per*_)/(*K*_*par*_ + *K*_*per*_). Specified anisotropy is positive (red, *K*_*par*_ > *K*_*per*_) in MID domain. (C) Resultant canvas shape (oblate spheroid). (D) Resultant canvas with major orientations of growth shown as lines. Lines are oriented parallel to the MID (sagittal) circumference (in contrast to Fig 4F). (E–H) Directional conflict resolution with anisotropy modulated by MID and STK. (E) Initial canvas showing domains of STK (green) and MID. (F) Initial canvas showing specified anisotropy is positive (red, *K*_*par*_ > *K*_*per*_) in MID domain and negative (blue, *K*_*par*_ < *K*_*per*_) in STK domain. (G) Resultant shape with slight indentation at STK region. (H) Midsection through resultant shape. (I–L) Directional conflict resolution with anisotropy modulated by MID, VEN, and STK. (I) Initial canvas showing VEN domain (magenta) as well as MID and STK. (J) Initial canvas showing specified anisotropy is positive (red, *K*_*par*_ > *K*_*per*_) in MID domain, enhanced (deeper red) in VEN domain, and negative (blue, *K*_*par*_ < *K*_*per*_) in STK domain. (K) The resultant shape is an oblate spheroid with elongated ventral midline that does not bulge out (contrast with Fig 4M). (L) Midsection through resultant shape (contrast with Fig 4N). (M–P) Integrated areal and directional conflict resolution. (M) Areal growth rates of integrated model in the initial canvas. Growth rate is promoted by MID (deeper orange midline) and inhibited by STK (white ‘South Pole’). (N) Initial canvas showing specified anisotropy is slightly positive (*K*_*par*_ > *K*_*per*_) in MID domain and enhanced (red) in broadened VEN domain. (O) The resultant shape is an oblate spheroid with elongated ventral midline that does not bulge out. (P) Midsection through resultant shape. Colour scale (B, F, J, N) is specified anisotropy. Models: http://cmpdartsvr3.cmp.uea.ac.uk/wiki/BanghamLab/index.php/Software and **https://doi.org/10.6084/m9.figshare.8966153.v1**, Models.7z archive. MID, Midline factor; STK, Stalk factor; VEN, Ventral factor; −ORG, minus-organiser; +ORG, plus-organiser.

To achieve specified anisotropy, we incorporated a polarity field into the model by introducing a source (plus-organiser [+ORG]) and sink (minus-organiser [−ORG]) of a propagating factor, polariser (POL). Taking the local gradient of POL allowed a polarity field to be specified. In a previous model of planar leaf development of *Arabidopsis*, the +ORG was positioned at the base of the leaf primordium, leading to a proximodistal gradient in POL [45]. By analogy, we introduced a +ORG in the stalk region of the *Utricularia* trap by having production of POL promoted by STK (Fig 6A). To provide a distal/marginal anchor for the polarity field, we also introduced a −ORG in the mouth region through enhanced degradation of POL (Fig 6A). Following a period of diffusion, POL concentrations were fixed to the initial canvas. Two local specified growth rates (specified strain rates) could then be defined: specified growth rate parallel to the polarity (*K*_*par*_) and specified growth rate perpendicular to the polarity (*K*_*per*_).

To generate the transformation from sphere to oblate spheroid, *K*_*par*_ was promoted by MID according to parameter *p*_*mid*_ (and *K*_*per*_ correspondingly reduced to keep specified areal growth rate at 3% h^−1^). The specified anisotropy, (*K*_*par*_ − *K*_*per*_)/(*K*_*par*_ + *K*_*per*_), is shown colour-coded in Fig 6B. This three-parameter model (*b*_*planar*_, *p*_*mid*_, *b*_*thickness*_) led to an oblate spheroid shape (Fig 6C, S6 Movie). The oblate spheroid was narrower than in the areal conflict model as the midline region grew less in width and was a better fit when compared to mature trap shape frontal (orange) and transverse (cyan) outlines. Also, in contrast to the areal conflict model, the maximal rate of resultant growth within the midline regions was oriented parallel to the midline rather than perpendicular to it (compare Figs 6D and 4F).

We next inhibited *K*_*par*_ by STK according to parameter *h*_*stk*_ (Fig 6E and 6F). This four-parameter model (*b*_*planar*_, *p*_*mid*_, *b*_*thickness*_, *h*_*stk*_) gave an oblate spheroid with a wide STK domain (Fig 6G and 6H, S7 Movie). The STK domain grew in width because of the high value of *K*_*per*_ needed to keep the total specified areal strain rate constant. To generate an elongated ventral midline, *K*_*par*_ was further promoted by VEN according to parameter *p*_*ven*_ (Fig 6I and 6J). This five-parameter model (interactions summarised in Fig 5B) gave an extended ventral midline region that was relatively straight compared to the bulged-out shape generated by the areal conflict model (compare Fig 6K and 6L, S8 Movie, with Fig 4M and 4N, S5 Movie). Thus, the directional conflict model accounted for the main shape transformations of the trap more effectively than the areal conflict model. However, the directional conflict model gave a less rounded STK domain than the areal conflict model and thus matched this aspect of development less effectively.

### Shape transformation through integrated areal and directional conflict resolution

To determine whether the various features could be captured with a single model, we developed an integrated model incorporating both directional and areal conflicts (seven-parameter model; interactions summarised in Fig 5C). To achieve this, we removed the constraint from the pure directional conflict model that specified areal growth rates were uniform. This allowed the specified areal growth rate in the stalk region to be lower than the rest of the canvas and enhanced in the midline, similar to the areal conflict model (compare Fig 6M with Fig 4L). We also broadened the domain of anisotropy in the VEN region (compare Fig 6N with Fig 6J), allowing for a better match to the cell-shape data described below (Fig 6P). These changes involved introducing two further parameters controlling specified growth rates. Running this model gave a shape showing a good match to that observed, with a small rounded STK domain at the final stage (Fig 6O and 6P, S9 Movie). A similar shape was generated if variation in rate of growth in trap wall thickness was incorporated, showing this did not have a marked effect (S3 Fig).Thus, the integrated model could account more effectively for observed trap shape transformations than models based purely on directional or areal conflict resolution (compare Fig 6O and 6P, S9 Movie, Fig 6K and 6L, S8 Movie with Fig 4M and 4N, S5 Movie).

Comparing the Growth regulatory network (KRN) of the integrated model (Fig 5C) with that proposed for *Arabidopsis* leaf development (Fig 5D, [45]) reveals both similarities and differences. In both cases, factors expressed in the midline region inhibit *K*_*per*_. In *Utricularia*, the factor is *VEN*, whereas in *Arabidopsis*, it is MID, which is expressed most strongly in the proximal midline. In both cases, *K*_*per*_ is low in the basal part, leading to a narrow supporting structure (stalk or petiole). In *Utricularia*, this is implemented by STK inhibiting *K*_*per*_, whereas in *Arabidopsis*, it is through a lamina factor (LAM) promoting *K*_*per*_. In *Utricularia*, STK also inhibits *K*_*par*_ because STK defines a domain intersecting the stalk and thus affects both its width and thickness.

A notable difference between *Utricularia* and *Arabidopsis* is that *K*_*par*_ is promoted in the midline regions of *Utricularia* (by VEN and MID) but not in *Arabidopsis*. This difference reflects the planar nature of *Arabidopsis* leaf growth. If the *Arabidopsis* midline region grew faster in length than the adjacent lamina, the midline would buckle out of the plane. In *Utricularia*, in which planarity is not required, enhanced growth of the midline regions leads to the oblate spheroid shape and increased length of the ventral midline. A further difference between the species is modulation of growth by a temporally varying factor (LATE) and a graded proximodistal factor (PGRAD) in *Arabidopsis*. In the absence of live imaging of regional growth in *Utricularia*, it is not clear whether such factors may also be involved in *Utricularia* trap development.

**Fig 7:**
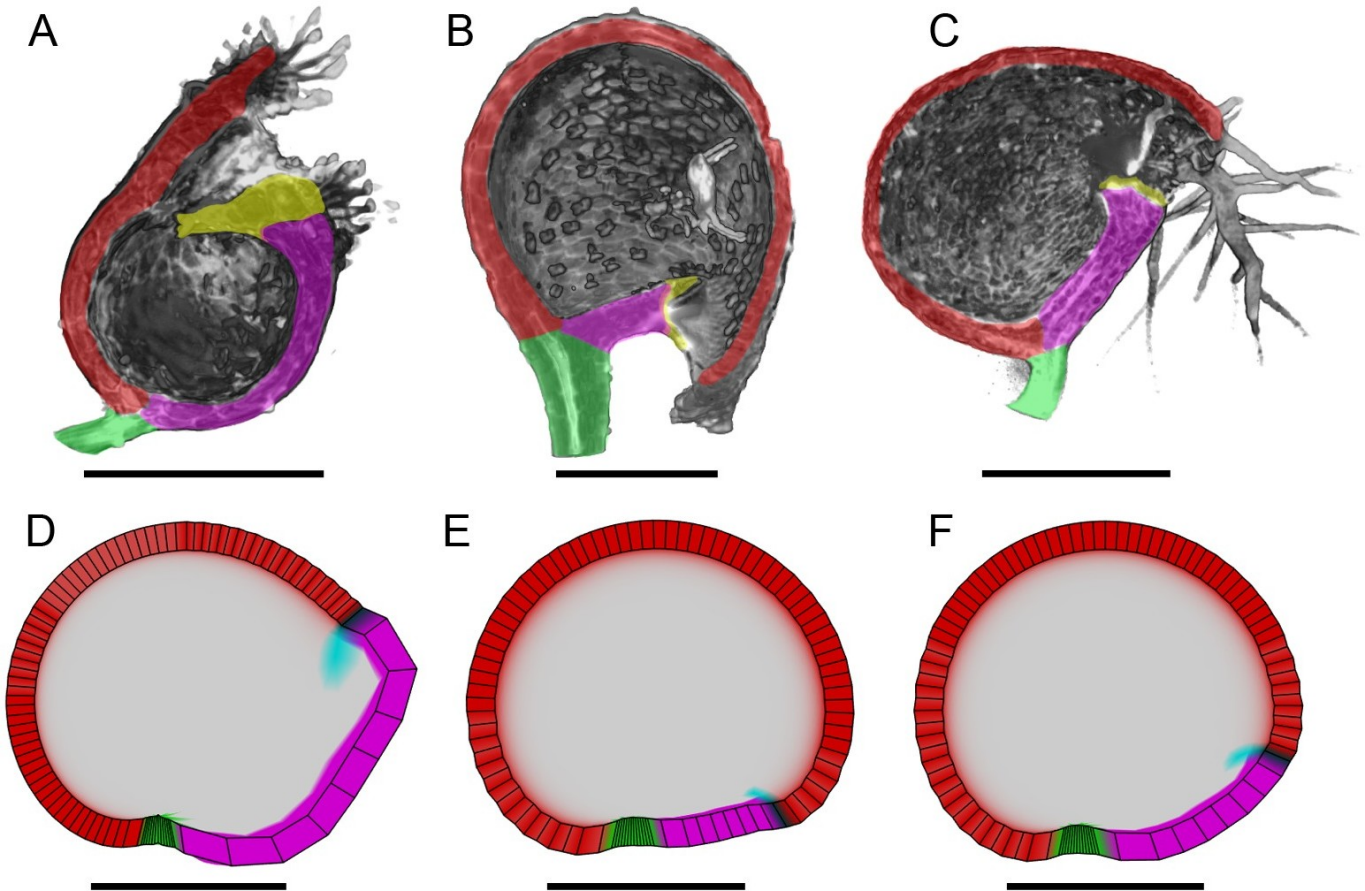
Trap shape variation between species. (A–C) Different traps from *Utricularia* species clipped in sagittal view. (A) *U*. *bisquamata* (terminal), (B) *U*. *praelonga* (basal), and (C) *U*. *gibba* (lateral). Dorsal midline (red), ventral midline (magenta), stalk (green), and threshold (yellow). (D–F) Illustrative modelling of different trap shapes, showing sagittal sections of resultant shapes. (D) Terminal type generated from integrated model by increased promotion of *K*_*par*_ by VEN and reduced promotion by MID. (E) Basal type generated from integrated model by decreasing promotion of *K*_*par*_ by VEN and increasing promotion by MID. (F) Lateral type, generated by integrated model as shown in Fig 6P. Scale = 500 μm. Data **https://doi.org/10.6084/m9.figshare.8966153.v1**, Fig 7.7z archive. Models: http://cmpdartsvr3.cmp.uea.ac.uk/wiki/BanghamLab/index.php/Software and **https://doi.org/10.6084/m9.figshare.8966153.v1**, Models.7z archive. MID, Midline factor; VEN, Ventral factor.

A further feature of the *U*. *gibba* model is that it allows evolutionary variation in trap shape to be explored. *Utricularia* traps vary in shape between species from terminal types, which have the mouth distant from the stalk (for example, *U*. *bisquamata*, Fig 7A), to basal types that have the mouth positioned near the stalk (for example, *U*. *praelonga*, Fig 7B) [46]. *U*. *gibba* belongs to a lateral type, intermediate between these extremes (Fig 7C). To illustrate how the model could be modulated to generate these forms, we varied the parameters by which MID and VEN affect *K*_*par*_. Increasing promotion of *K*_*par*_ by VEN and reducing promotion by MID gave a shape resembling the terminal type (Fig 7D), whereas decreasing promotion of *K*_*par*_ by VEN and increasing promotion by MID gave the basal type (Fig 7E). The resultant shape of the integrated model is closest to the *U*. *gibba* intermediate type (Fig 7F). The extent to which such variations are valid could be tested by analysing the growth of each trap type.

### Cellular-level data and modelling

To explore how cell growth and division may be integrated within these models, we tiled the canvas with virtual cells (polygons) with vertices that are displaced as the canvas grows [6]. New walls may then be introduced through cell division as cells reach a threshold size. In this modelling framework, cell divisions do not contribute to specified growth rate of the tissue but respond to the pattern of local growth. This is consistent with the mechanism of plant cell growth, which is driven by turgor pressure stretching the cell walls. Cell divisions thus provide new partitions that maintain the material properties of the growing cell-wall mesh rather than being drivers of growth.

**Fig 8:**
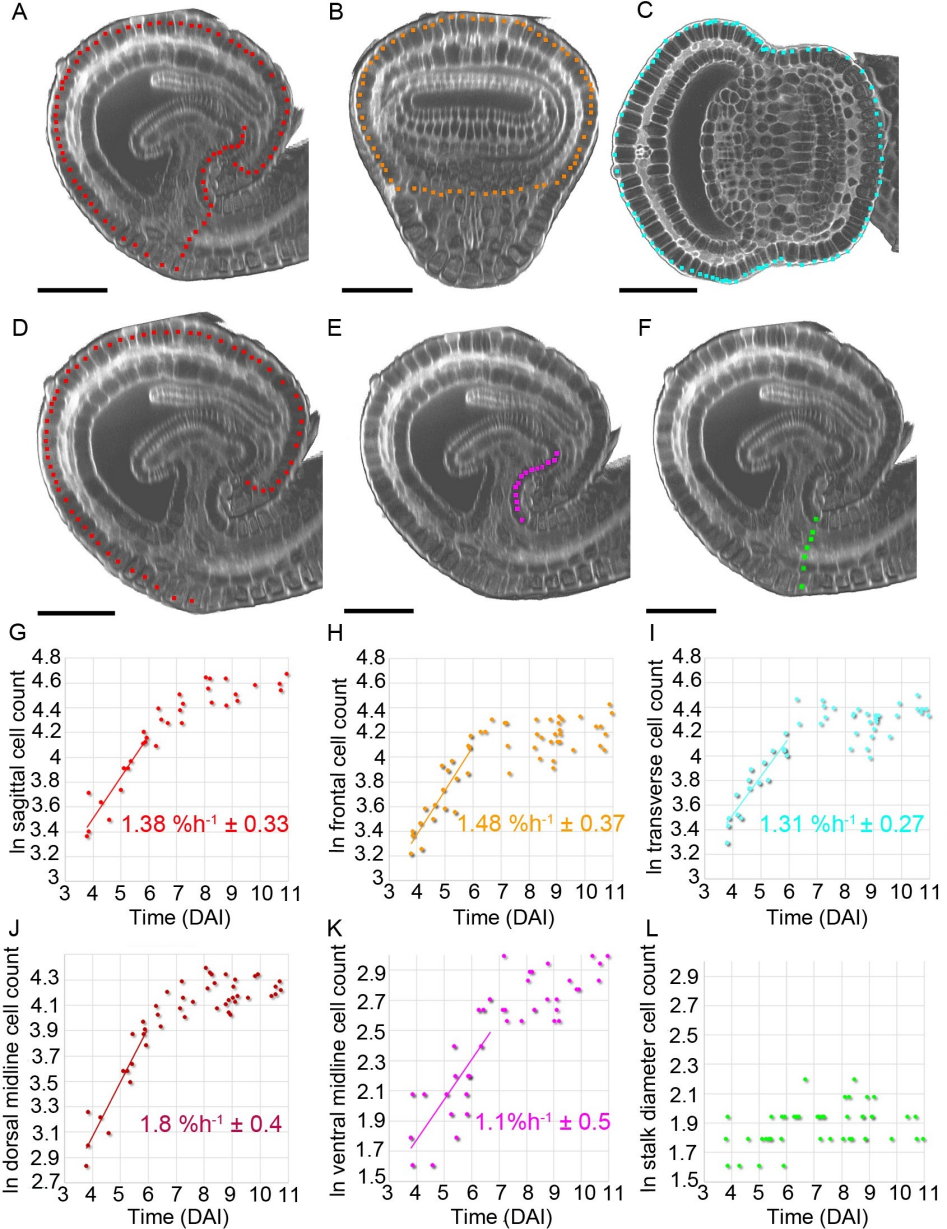
Cell counts at different stages of trap development. (A–C) *U*. *gibba* traps between 3 and 11 DAI (live and PI-stained) were imaged with a confocal microscope and clipped in VolViewer. Circumference cell counts were obtained by manually placing points in (A) sagittal (red points), (B) frontal (orange), and (C) transverse (cyan) planes. In mature traps, where it was not possible to image the entire depth of the trap by confocal microscopy, half the circumference cells were counted in frontal and transverse views. This value was doubled to obtain estimated cell numbers. Trap shown was 139 μm long, 6.1 DAI. Scale bar = 50 μm. (D–F) Sagittal shown in (A) illustrating regional cell counts: (D) dorsal midline (red), (E) ventral midline (magenta), and (F) stalk diameter (green). (G–L) Natural log of cell number for the regions indicated above (A–F) plotted against time (DAI) and trend lines fitted during the early exponential period, S5 Data. Slopes (percent increase in cell number per hour) and twice the standard deviation of the slopes indicated. Note that this value may be less than the strain rate, in which case cell size increases as well as cell number. (G) Sagittal circumference, R^2^ = 0.8635, *n* = 13 traps. (H) Frontal circumference, R^2^ = 0.8908, *n* = 19 traps. (I) Transverse circumference R^2^ = 0.8602, *n* = 19 traps. (J) Dorsal midline, R^2^ = 0.8701, *n* = 14. (K) Ventral midline, R^2^ = 0.5496, *n* = 18. (L) Stalk cell number did not increase. Traps showed 5.78% ± 0.45 shrinkage when prepared for OPT (S9 Data). To compensate for this, trap-length measurements of all fixed traps were increased by 5.78% before time (DAI) calculation. Data **https://doi.org/10.6084/m9.figshare.8966153.v1**, Fig 8.7z archive. DAI, days after initiation; OPT, Optical Projection Tomography; PI, propidium iodide.

To provide constraints for such cellular-level models, we first estimated cell division parameters by counting cell numbers along different circumferences and regions (Fig 8A–8F). For all regions except the stalk, cell numbers showed an approximately exponential increase until about 6–7 DAI, after which cell numbers levelled off (Fig 8G–8L, S5 Data). This suggests an early phase when cell divisions occur, followed by a later phase after 6–7 DAI, when cell division slows down or arrests. During the division phase, the rate of increase in cell number was 1%–2% h^−1^, comparable to the strain rates (Fig 3K and 3L, S3 Data), suggesting cell division broadly keeps up with growth. The reduced rate of division after 6–7 DAI did not correlate with a change in growth rate, which reduced later (approximately 11 DAI, Fig 3K and 3L, S3 Data), consistent with division not being the driver of growth. Estimates of cell area during the division phase gave a mean of approximately 50 μm^2^ (S4 Fig, S6 Data). The stalk region showed very little change in cell number, indicating that cell division rates were low in this region over the period analysed (Fig 8L, S5 Data). This lack of division correlated with a slow growth rate.

Cellular-level models were developed according the principles established in the analysis of *Arabidopsis* leaf development [6] but extended from a flat sheet to a curved sheet embedded in three dimensions. The surface of the initial canvas was tiled with an array of cells with mean cell area of 50 μm^2^ (approximately 350 cells). As in *Arabidopsis*, we invoked a dual-control model in which there is spatiotemporal regulation of both growth (*K*_*par*_ and *K*_*per*_) and cell division. Cell division required expression of a factor conferring division competence (CDIV) that was expressed throughout the canvas (except in the STK region) until 6.5 DAI, after which it was switched off. Execution of division occurred when cells reached a threshold area, *T*_*A*_, set to 70 μm^2^. Assuming symmetric division, this would give daughters of 35 μm^2^ and a mean cell area of approximately 50 μm^2^. Thus, incorporating cells involved two additional parameters, both of which were experimentally constrained: mean division threshold *T*_*A*_ = 70 μm^2^ and inactivation of CDIV in all cells at 6.5 DAI. For simplicity, the mouth region was modelled in a similar way to the rest of the tissue and is shown in grey in output images. Cell sizes and cell shapes were an emergent property that resulted from running the model.

**Fig 9:**
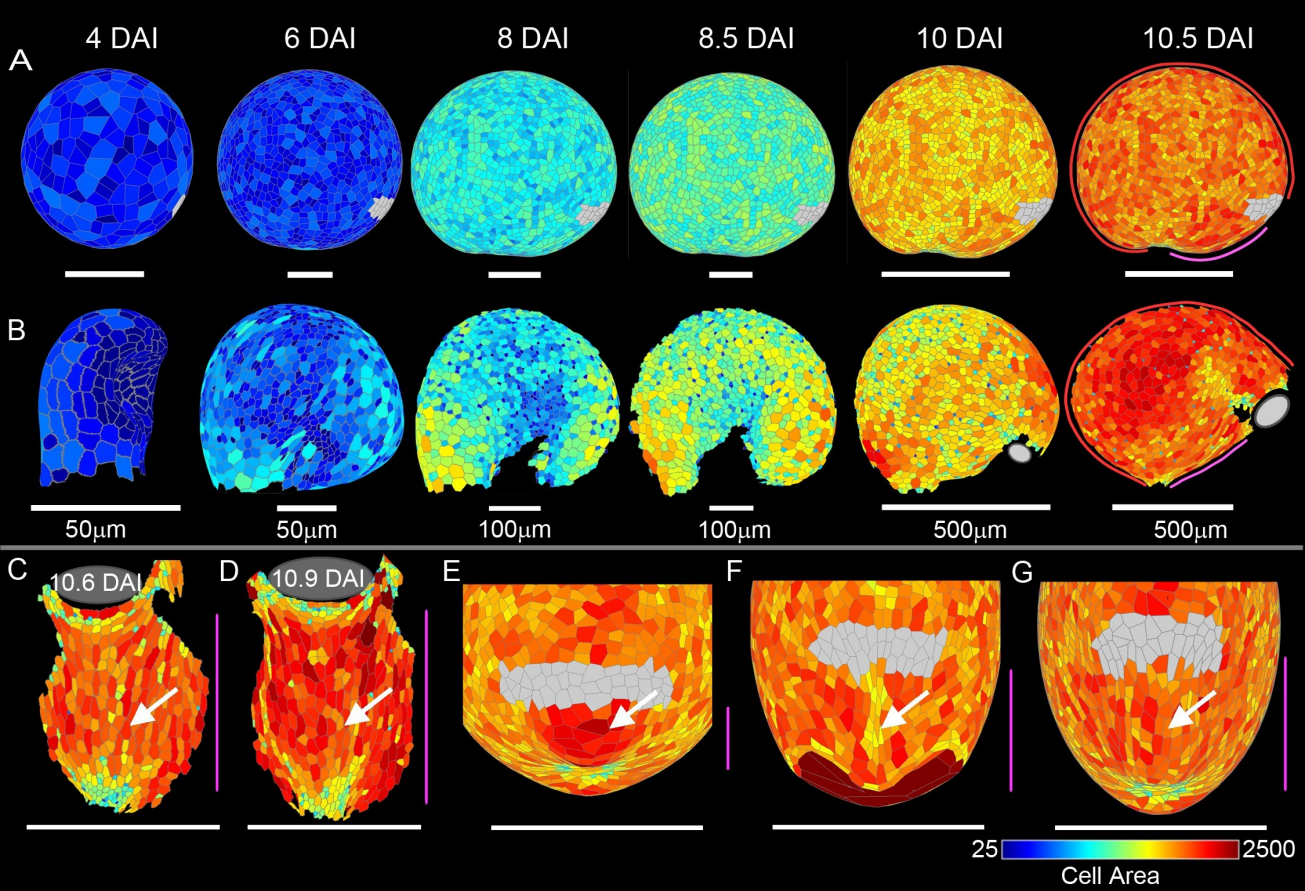
Cellular-level area models and data. (A) Growth snapshots of integrated areal and directional conflict model, side view from 4 DAI canvas start shape to resultant canvas shape at 10.5 DAI, coloured for cell area. Grey region shows approximate location of mouth. (B) Experimental data showing traps with cells segmented and coloured for cell area at time points corresponding to those shown for the integrated model shown in A, side view. Grey region shows approximate location of mouth where visible. (C–D) Experimental data. (C) Trap with cells segmented and coloured for cell area, front view; arrow highlights ventral midline cells. (D) Additional segmented data, arrow highlights ventral midline cells. (E–G) Zoomed-in resultant model front views. (E) Areal conflict model; arrow highlights larger ventral midline cells. (F) Directional conflict model; arrow highlights smaller ventral midline cells. (G) Integrated model; arrow highlights ventral midline cells. Magenta line shows ventral midline, and red line shows dorsal midline. In all images, colour scale shows cell area (μm^2^) on logarithmic scale. Data **https://doi.org/10.6084/m9.figshare.8966153.v1**, Figs 9, 10, S4 and S6.7z archive. Models: http://cmpdartsvr3.cmp.uea.ac.uk/wiki/BanghamLab/index.php/Software and **https://doi.org/10.6084/m9.figshare.8966153.v1**, Models.7z archive. DAI, days after initiation.

Running the areal, directional and integrated models with these assumptions gave cell areas in the main trap of less than 70 μm^2^ until 6.5 DAI, after which cell size increased following division arrest (integrated model shown Fig 9A, S10 Movie, S11 Movie [other models in S5 Fig, S12 Movie and S13 Movie]). Cells in the STK region enlarged from an earlier stage because they did not divide because CDIV was absent.

To evaluate these models against experimental data, we segmented 3D confocal images of traps at corresponding stages to those shown for the model (Fig 9B). The observed cell sizes showed more variation in spatial pattern than generated by our simplified models, indicating that more elaborate mechanisms operate for spatiotemporal control of division and/or growth than those implemented. Nevertheless, broad trends could be compared. As with the model outputs, cell area increased after 6–7 DAI (S4 Fig, S6 Data), except for a subgroup of cells (hemispherical gland cells) that remained small (S6 Fig). Final cell areas in the experimental data showed no major enhancement or reduction in cell areas in the ventral midline (arrowed in the two examples of Fig 9C and 9D). By contrast, in the areal conflict model, cells were larger in the midline regions because these were the regions of higher growth rate (arrowed in Fig 9E). In the directional conflict model, cells along the ventral midline were smaller (arrowed in Fig 9F). This reduction in cell size arises through the directional conflict, which leads to reduced resultant areal growth rate in this region throughout the simulation. This effect was absent in the integrated model because *K*_*per*_ was reduced by less and over a broader domain (arrowed in Fig 9G). Thus, the integrated model gave the best overall match to the pattern of cell sizes in this region (compare Fig 9G to Fig 9C and Fig 9D).

**Fig 10:**
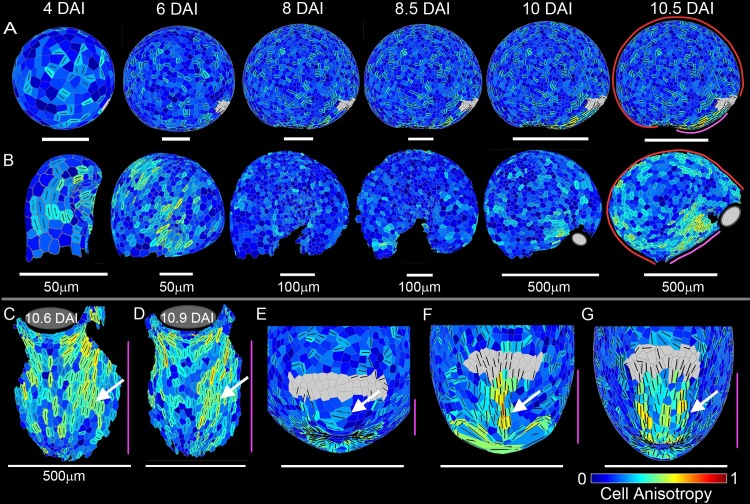
Cellular-level anisotropy models and data. (A) Integrated areal and directional conflict model side view from 4 DAI canvas start shape to resultant canvas shape at 10.5 DAI, coloured for cell anisotropy. Lines show orientation of the cell long axis and are shown where anisotropy exceeds 0.23. Grey region shows approximate location of mouth. (B) Experimental data showing traps with cells segmented and coloured for cell anisotropy at time points corresponding to those shown for the integrated model shown in (A), side view. Grey region shows approximate location of mouth where visible. (C–D) Experimental data; arrows highlight region of cell anisotropy parallel to the ventral midline. (C) Trap with cells segmented and coloured for cell anisotropy, front view. (D) Additional segmented data. (E–G) Zoomed-in resultant model front views. (E) Areal conflict model; arrow highlights region of cell anisotropy perpendicular to the ventral midline. (F) Directional conflict model; arrow highlights region of cell anisotropy parallel to the ventral midline. (G) Integrated model; arrow highlights wider region of cell anisotropy parallel to the ventral midline. Magenta line shows ventral midline; red line shows dorsal midline. Grey region shows mouth. Colour scale shows cell anisotropy. In all images, cell-shape anisotropy is defined by R − 1/R + 1, where R is the ratio of the long/short axis of an ellipsoid fitted to the cell. This equation evaluates to 0 for isometric cell shape and 0.333 when the long axis is twice the short axis. Data **https://doi.org/10.6084/m9.figshare.8966153.v1**, Figs 9, 10, S4 and S6.7z archive. Models: http://cmpdartsvr3.cmp.uea.ac.uk/wiki/BanghamLab/index.php/Software and **https://doi.org/10.6084/m9.figshare.8966153.v1**, Models.7z archive. DAI, days after initiation.

Another possible test of the models might be the pattern of cell-shape anisotropy because the models make very different predictions. The areal conflict model predicts resultant growth anisotropy perpendicular to the midline (Fig 4F), whereas the directional conflict model predicts resultant anisotropy parallel to the midline, particularly in the ventral midline (Fig 6D). These anisotropies in growth will affect cell shape after cell divisions cease. To evaluate these effects, we colour-coded the cells generated by models according to their cell-shape anisotropy (defined as R − 1/R + 1, where R is the ratio of the long/short axis of an ellipsoid fitted to the cell) and showed the orientation of the cell long axis with a black line for cells with strong anisotropy (results for integrated model shown in Fig 10A, other models in S7 Fig). To allow direct comparison with the experimental data, we used the same colour-coding system for the segmented cells of traps (Fig 10B).

The most striking region of high anisotropy in the experimental data was in the ventral region, where the long axis of the cells was oriented mainly parallel to the ventral midline (arrowed in the two examples showing in Fig 10C and 10D). This finding was inconsistent with output of the areal conflict model (Fig 10E, S14 Movie) but was predicted by the directional conflict model (Fig 10F, S15 Movie). An even better match with the experimental data was obtained with the integrated model (Fig 10G, S16 Movie, S17 Movie), which invoked a broader domain of specified anisotropy in the ventral midline.

In principle, further modifications and parameters could be incorporated to the integrated model to give a better match to the distribution of cell patterns or shape of the trap. Additional features, such as introducing a discontinuity at the mouth and simulating its opening at later stages, could also be introduced. However, without further experimental data to test and constrain the modelling, such an exercise may not be mechanistically informative.

### Clonal analysis

Cell-shape analysis provides evidence for growth anisotropy after cell divisions have ceased (after 6–7 DAI). To determine whether growth anisotropy was also present during earlier phases, when divisions were occurring, we used clonal analysis to examine the size and shape of patches of cells generated by single progenitor cells. We achieved this by developing a transformation protocol for *U*. *gibba* (detailed in Materials and Methods) and introducing a heat-shock (HS)–inducible Cre-lox system generating clones expressing GFP on an mCherry background.

**Fig 11:**
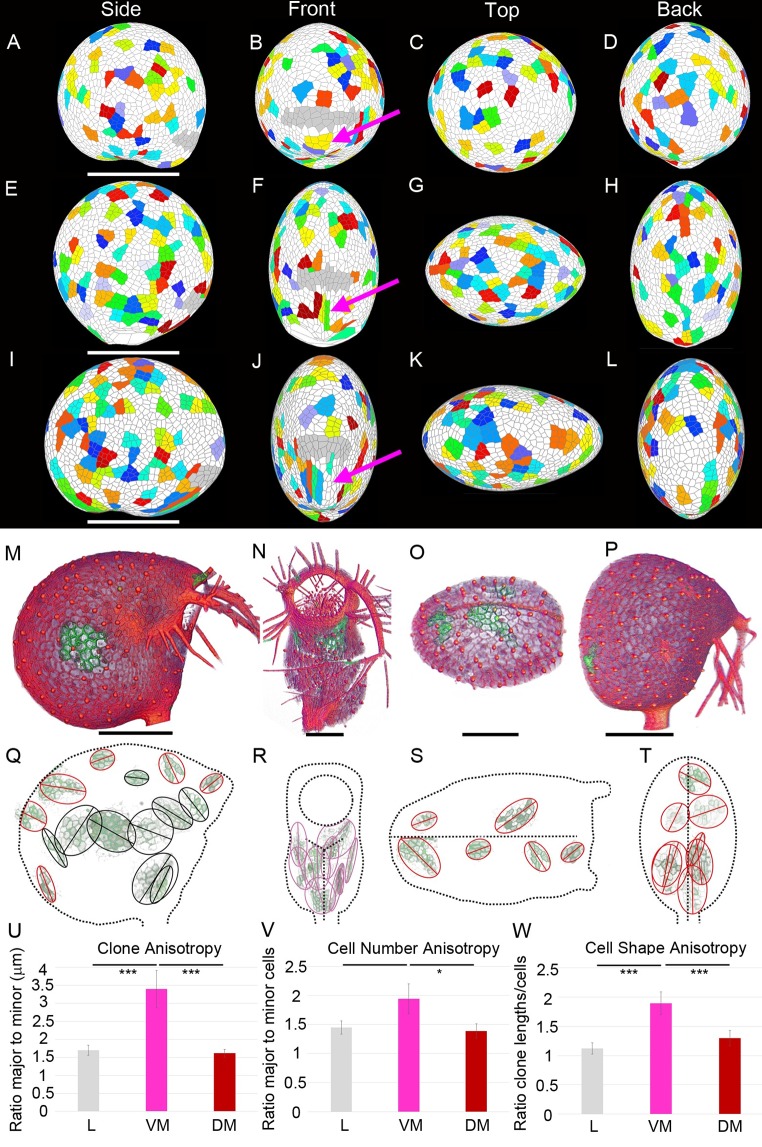
Clonal analysis. (A–D) Virtual clones generated by areal conflict model. Clones were induced at 4 DAI. Resultant model outputs shown are 10.5 DAI. Magenta arrows highlight ventral midline clones. (A) Side view, (B) front view, (C) top view, (D) back view. Scale bar 500 μm. (E–H) Virtual clones generated by directional conflict model. Scale bar 500 μm. (I–L) Virtual clones generated by integrated areal and directional conflict model. Scale bar 500 μm. (M–P) HS-induced clones (green) imaged with a confocal microscope at 10–11 DAI. Scale bars 250 μm. (Q–T) Sector images were placed in their approximate location on the trap (dashed outlines). Ellipses were fitted to sectors, and major axes are shown (S17 Data). (U–V) Data histograms (S7 Data). (U) Clone anisotropy. Ratio of major/minor axis lengths for clones, ± SE. *P*-values of *t* tests are L to VM *p* = 0.0004 (***), VM to DM *p* = 0.0007 (***), DM to L *p* = 0.65. (V) Cell-number anisotropy. Ratio of cell numbers along major/minor axes of clones, ± SE. *P*-values of *t* tests are L to VM *p* = 0.053, VM to DM *p* = 0.046 (*), DM to L *p* = 0.73. (W) Cell-shape anisotropy. Ratio of clone anisotropy/cell-number anisotropy for individual clones, ± SE. *P*-values of *t* tests are L to VM *p* = 8.56 × 10−^5^ (***), VM to DM *p* = 0.0003 (***), DM to L *p* = 0.8; *N* = 59 clones in 36 traps. L = 25, VM = 15, DM = 19. Data **https://doi.org/10.6084/m9.figshare.8966153.v1**, Fig 11.7 archive. Models: http://cmpdartsvr3.cmp.uea.ac.uk/wiki/BanghamLab/index.php/Software and https://doi.org/10.6084/m9.figshare.8966153.v1, Models.7z archive. DAI, days after initiation; DM, dorsal midline; HS, heat shock; L, lamina; VM, ventral midline.

We first analysed the pattern of the shapes of clonal regions generated by each model. Virtual clones were generated by colour-coding approximately 40% of cells at approximately 4 DAI and following their descendants (Fig 11). Virtual clones could have two components contributing to their overall anisotropy in shape. First, there could be more cells along the major axis of the clone (cell-number anisotropy), arising because of anisotropic growth during the period when cell divisions occur. Second, cells could be elongated along the major axis of growth (cell-shape anisotropy), arising because of anisotropic growth after division arrest. The anisotropy of the clone as whole (clone anisotropy) is the product of cell-number and cell-shape anisotropy. In the directional and integrated models, both types of anisotropy contribute to the elongated clones of the ventral midline region (Fig 11F and 11J arrowed, S18 Movie, S19 Movie [as compared to the areal model, S20 Movie]).

To compare these predicted patterns to clones generated experimentally, we induced clones using the Cre-lox system [47]. We introduced a construct with the cauliflower mosaic virus (CaMV) 35S promoter driving GFP interrupted by an mCherry coding sequence with a terminator, flanked by lox recombination sites (see Materials and Methods). The construct also carried Cre recombinase under the control of an HS promoter. Following HS, GFP sectors were visualised 4 days later, when the trap size was at that expected for approximately 10 DAI. Clones between 3–30 cells were selected for measurement. Fig 11M–11P (and S21 Movie, S22 Movie) illustrate some of the sectors obtained. A summary of results for clones in the different regions of the trap are shown in Fig 11Q–11T, (S17 Data). In the ventral midline region, clones were preferentially oriented parallel rather than perpendicular to the midline, consistent with the directional conflict and integrated models.

To quantify these components of anisotropy in the experimental data, we first subdivided the trap into ventral midline, dorsal midline, and lamina domains. A clone was considered to be within the midline domain (dorsal or ventral) if its centre was a distance of five cells or less from the midline. For each domain, we measured the ratio of the long/short axis of each clone (clone anisotropy), the ratio of cell number along the long axis and short axis of each clone (cell-number anisotropy), and clone anisotropy divided by cell-number anisotropy (cell-shape anisotropy). Clone anisotropy was significantly higher in the ventral midline region compared to the other regions (Fig 11U, S7 Data). Some of this difference came from cell-number anisotropy, which was significantly higher in the ventral compared to dorsal midline regions (Fig 11V, S7 Data). Cell-shape anisotropy was also significantly higher in the ventral midline region (Fig 11W, S7 Data). These results are more consistent with the directional conflict and integrated models than the areal conflict model (compare Fig 11E–11H with Fig 11I–11L and Fig 11A–11D and S18 Movie, S19 Movie, and S20 Movie) and indicate that anisotropic growth occurred in the ventral midline region both during the phase of cell division and after cell division arrest.

### Evidence for a polarity field

**Fig 12:**
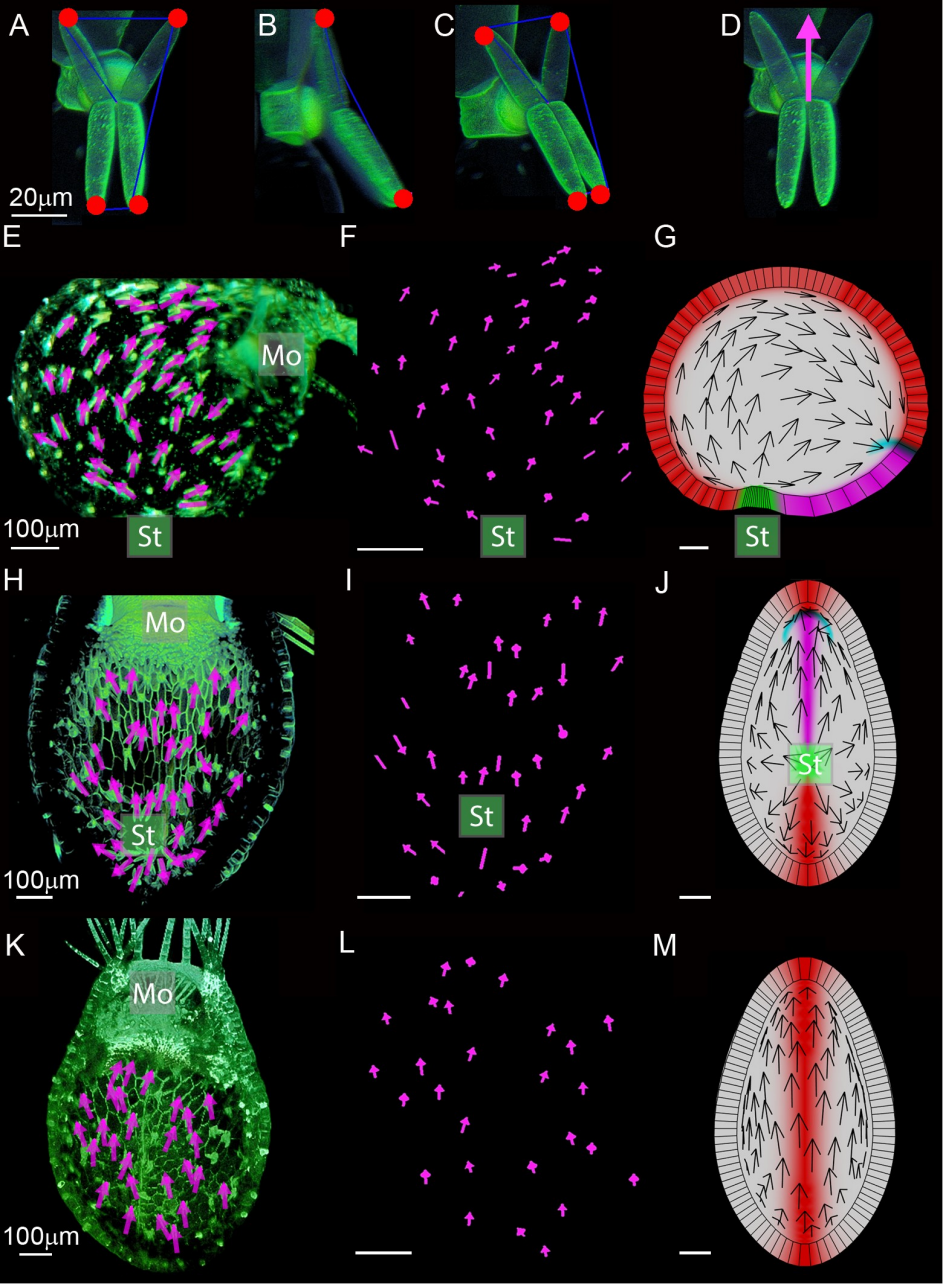
Evidence for a polarity field in traps. (A–D) Points at the quadrifid gland centre and at ends of each quadrifid gland arm were placed in VolViewer (red spots). Arrows (example shown in D) were oriented toward the greatest distance between arms with quadrifidScript software (DistArms). (E–G) Clipped sagittal view of OPT scan, looking into the trap at quadrifid glands on one side of the trap. (E) Arrows flow from stalk (St) to mouth (Mo). Lines with no arrow heads were allocated when the difference in distance between arms was less than a threshold value of 2 μm. Arrows were enlarged in Adobe Illustrator for clarity. (F) DistArms polarity arrow output from VolViewer shown in (E) displayed alone. 35/37 glands in proximodistal orientation, five unallocated (S8 Data). (G) Output of integrated model clipped sagittal view. Arrows indicate tissue polarity field from stalk to mouth. Stalk (green), mouth (cyan), dorsal midline (red), ventral midline (magenta). (H–J) Transverse clipped views of confocal scan looking into the ventral half of the trap. (H) Arrows diverge at stalk (St) and flow from stalk to mouth (Mo). (I) Polarity arrows shown in (H) displayed alone. 29/32 glands point away from stalk (S8 Data). (J) Output of integrated model, transverse clipped view into trap towards stalk. Shows diverging tissue polarity field from stalk to mouth. (K–M) Transverse clipped view of confocal scan looking into the dorsal half of the trap. (K) Polarity arrows shown in (K) displayed alone. 27/27 glands point to mouth (S8 Data). (L) Arrows point towards the mouth. (M) Output of integrated model, clipped view into top of trap. Arrows point towards mouth. Data **https://doi.org/10.6084/m9.figshare.8966153.v1**, Fig 12 and S8.7z archive. Models: http://cmpdartsvr3.cmp.uea.ac.uk/wiki/BanghamLab/index.php/Software and **https://doi.org/10.6084/m9.figshare.8966153.v1**, Models.7z archive. OPT, Optical Projection Tomography.

The above models involving directional conflicts hypothesised a polarity field running from stalk to mouth. To test whether such a polarity field exists, we analysed the pattern of glands on the inside of the trap because hair morphology has been used to infer cell polarity fields in several cases. As previously noted [13], quadrifid glands, which decorate the inside of the mature trap, often have arms more splayed out at one end than the other (Fig 12, S23 Movie).

To determine whether this polarity of the quadrifids is organised as a field, the glands were imaged in three dimensions using OPT and confocal microscopy. Five landmark positions were identified on each quadrifid, one at the centre and one at each arm tip (Fig 12A–12C). The landmarks were placed by examining each quadrifid in isolation. The two pairs of nearest tip landmarks were identified and the distances between them calculated and subtracted from one another. The quadrifid was assigned a polarity, pointing towards the more widely splayed arms and passing through the middle of the quadrifid (Fig 12D, arrow). A threshold value was applied to identify glands in which polarity could not be assigned with confidence. In these cases, quadrifid was assigned an axiality (line passing through the middle of the quadrifid) but no polarity. Quadrifid polarities and axialities were viewed in three dimensions (S24 Movie, S25 Movie) or projected in 2D (Fig 12). An additional measure was applied to confirm that quadrifid polarities based on distance between arms was consistent with relative arm lengths (S8 Fig).

In side views, quadrifid polarities mainly pointed away from the stalk region and towards the mouth (for example, 35/37 in the example shown in Fig 12E and 12F, S24 Movie, S8 Data, another example shown in S8D Fig), consistent with the hypothesised polarity field (Fig 12G). Ventral (Fig 12H and 12I, S25 Movie, S8G Fig, S8J Fig) and dorsal views (Fig 12K and 12L, S8M Fig, S8P Fig) also indicated polarity running from stalk to mouth, consistent with the proposed polarity field (Fig 12J and 12M). The sense in which the polarity arrow points is arbitrary and could equally well be depicted as going from mouth to stalk. The key observation is that the polarity is coordinated to point preferentially in one direction over the other. Thus, the quadrifid analysis supported the polarity field hypothesised in the model.

## Discussion

We show that following formation of a near-spherical shape at early stages of development, the *Utricularia* trap flattens to form an oblate spheroid and develops an extended ventral midline. We further show that this shape change, as well as the broad pattern of cell shapes and sizes, can be accounted for by a model that invokes many of the same principles as those used to account for *Arabidopsis* leaf development but in the context of a highly curved tissue sheet. Our results thus indicate that simple modulations of core developmental processes can account for shape changes in a diverse range of contexts.

The model we propose has experimentally constrained parameters influencing specified growth rates (parallel or perpendicular to a polarity field) and affecting cell division (threshold area for division execution and duration of division competence). Changes in organ curvature and cell sizes and shapes are not directly specified but emerge from running the model because of mechanical connectivity between regions. By introducing different elements into the model progressively and comparing outputs to experimental observations, the contribution of particular components could be evaluated.

### Organisation of polarity field

To account for *Utricularia* trap morphogenesis, we invoked a proximodistal polarity field to provide orientational information for specified anisotropic growth (Fig 6). The polarity field radiates out from a +ORG at the base (stalk) and converges distally towards a −ORG near the mouth region. The field is comparable to that proposed for *Arabidopsis* leaf development, except that instead of propagating on a flat sheet, it propagates through a curved sheet. The polarity field is parallel to the midline in both cases. However, in contrast to the *Arabidopsis* leaf, in which the midline is a linear continuation of the petiole, the midline of the *Utricularia* trap forms a curve that traverses the stalk, reflecting its peltate organisation [16].

Evidence for the proposed polarity field in *Utricularia* comes from the analysis of quadrifid glands. These glands can be used as indicators of polarity, similar to hairs in *Drosophila* wing [48] or trichomes on leaves of *Arabidopsis* or barley [49–51]. The coordinated polarity exhibited by these hairs, glands, or trichomes likely reflects polarity fields established much earlier in development, for example, by planar or tissue cell polarity pathways [43,52–57].

In our model, we propose that a key role of the polarity field in *Utricularia* is to coordinate the orientation of specified anisotropic growth. Alternatively, specified anisotropic growth could be coordinated through the orientation of stresses [58]. Stresses can originate from differential growth of a tissue sheet (areal conflict), and it has been proposed that such residual stresses might change local cell-wall properties to orient specified anisotropic growth without the need for a polarity field [59]. However, computer simulations show that such a model does not allow local orientations to be specified in a stable manner [41,59]. Another possibility is that differential pressure within the trap generates circumferential stresses that orient growth. In normal conditions, the interior of the mature trap is under low pressure caused by water being pumped out of the trap chamber [36,60,61]. The differential pressure could create circumferential stresses that orient growth. However, under the tissue culture conditions employed for the growth of *Utricularia* here, the traps develop with little pressure differential (their shape is similar to the relaxed state), making this mechanism unlikely.

### Contributions of directional and areal conflict resolution

We show that both areal and directional conflict resolution play a role in shaping the *Utricularia* trap. A role for areal conflict resolution is suggested by the slow growth rate in diameter of the stalk. A role for directional conflict is suggested by tissue-level modelling: generation of an extended ventral midline is most readily achieved through greater specified growth parallel compared to perpendicular to the midline (i.e., anisotropic-specified growth). We found that with isotropic-specified growth alone, the ventral midline bulges out. Further support for specified anisotropy derives from clonal analysis, which indicates greater cell proliferation parallel compared to perpendicular to the ventral midline. Cell-shape analysis also shows that cells are more elongated parallel to the ventral midline, consistent with anisotropic growth after the cessation of division. Enhanced growth parallel to polarity along the midvalve region of *Capsella* fruits (which corresponds to the midline of the carpel primordia) has also been proposed to account for shape change [62].

Comparisons between the growth model of the *Utricularia* trap and that proposed for *Arabidopsis* leaf development reveal both similarities and differences. In both cases, specified growth perpendicular to the polarity is inhibited in the midline and proximal domain, leading to a narrow midline and supporting structure (stalk or petiole). However, growth parallel to the polarity is promoted in the midline regions of *Utricularia* but not in *Arabidopsis*. This difference reflects the planar nature of *Arabidopsis* leaf growth. If the *Arabidopsis* midline region grew faster in length than the adjacent lamina, the midline would buckle out of the plane. In *Utricularia*, where planarity is not required, enhanced growth of the midline regions leads to the oblate spheroid shape and increased length of the ventral midline.

### Cell division and growth

During the early phases of *U*. *gibba* trap morphogenesis, cell division occurs concurrently with growth, whereas at later stages, growth occurs in the absence of division, leading to cell expansion. This is comparable to the situation in planar leaf development [63]. We model cell division in the *U*. *gibba* trap through a dual-control mechanism in which both cell division and growth parameters are under spatiotemporal regulation. We assume cells are competent to divide at early stages (before 6.5 DAI), after which divisions are arrested. Competent cells execute division when they reach a threshold size. Such a model broadly accounts for observed clonal anisotropy in both cell number and cell shape in clones. Anisotropy in cell number arises through anisotropic growth during the cell division phase, whereas anisotropy in cell shape arises through anisotropic growth after divisions have ceased. This type of model contrasts with that proposed for *Sarracenia* pitcher leaf development, in which changes in the planes of cell division are proposed to drive the development of leaf shape [64]. In our model, the planes of cell division follow as a result of differentially oriented growth rather than being the primary cause of morphogenesis.

### Limitations of the model

The model we present aims to capture overall external shape change of the trap but does not account for the more subtle patterns of spatiotemporal variation in cell sizes and shapes of the trap. It also does not account for formation of the mouth opening or the internal foldings and thickenings that occur within the trap, generating the threshold and trap door (Fig 1B and 1C). The model considers only the later stages of morphogenesis after the trap is a near-spherical shape. Curvature of the trap primordium is present before this stage, appearing as soon as the sheet-like nature of the primordium becomes evident. This suggests that sheet formation, which depends on control ab/adaxial patterning in planar leaves [4], is intimately linked with formation of the spherical shape. Further hypothesis development, including the modelling of sheet formation [65] and experimental testing, may be feasible in *U*. *gibba*, given its small genome and potential as a model system for carnivorous plants [66].

### Evolution of leaf shape

The shape of *Utricularia* traps is highly constrained by the need to have a sealed trap door that allows low pressure to be established within the trap lumen and released upon triggering [27,36,37,67]. Despite this constraint, *Utricularia* traps vary in shape between species from terminal types, which have the mouth distant from the stalk, to basal types that have the mouth positioned near the stalk [46]. *U*. *gibba* belongs to a lateral type, intermediate between these extremes. We show that a simple mechanism for generating these trap morphologies is to vary the relative rates of growth along the ventral and dorsal midlines, with basal types having the lowest and terminal types the highest ratios of ventral to dorsal growth. If specified growth was purely isotropic, changing these ratios may have consequences on trap function by modifying trap curvature around the midlines. Our analysis shows that orienting anisotropic growth through a polarity field may allow such changes in curvature to be controlled by varying the extent of growth parallel and perpendicular to the polarity. Thus, the morphogenetic system described here may provide great flexibility in allowing shape to evolve even when under strong functional constraints.

## Materials and methods

### Plant material and specimen preparation

*U*. *gibba* (The Fly Trap Plants, Bergh Apton, UK) was grown in a heated glasshouse set to 22°C in plastic trays with 3 cm 1/1 peat/silver-sand mix, topped up with 500 ml deionised water. After flowering, collected seeds were surface sterilised for in vitro culture in 70% ethanol, 0.1% SDS solution for 5 minutes; washed in water, 1 × 4% parazone bleach, 0.2% triton X- 100 (11332481001, Merck, Darmstadt, Germany) treatment for 10 minutes; and washed three times in sterile water. Seeds were germinated in sterile pots (100 ml Sterilin jar, 185AM; Slaughter, Basildon, UK) with 25 ml of solid 1B culture medium (2.2 g/l MS (Murashige and Skoog Medium Mod. No. 1B, M0233; Duchefa Biochemie, Haarlem, the Netherlands), 2.5 g/l sucrose, 1.5 g/l Gelrite agar (Gelzan CM, G1910 [pH 5.8]; Merck) topped up with 30 ml liquid 1B medium with ethephon (2.2 g/l MS, 2.5 g/l sucrose [pH 5.8], 0.1 mM ethephon [C0143, Merck]). Pot lids were sealed with Micropore tape. After germination, plants were transferred to liquid 1B medium (2.2 g/l MS, 2.5 g/l sucrose [pH 5.8]). In vitro cultured plants were grown in a controlled environment room at 25°C, with 16-hour light and 8-hour dark cycles, and subcultured every two weeks.

### Growth tracking

A cut piece of *U*. *gibba* stolon approximately 3 cm long from in vitro culture was placed in liquid 1B medium in a small Petri dish (Sterlin, 50-mm diameter, 124; Slaughter). The youngest trap after emergence from the circinate apex (approximately 150 μm) was imaged every 24 hours until maturity under bright-field light on a Leica M205C stereomicroscope with a Leica DFC495 camera (Leica, Milton Keynes, UK). Trap length (Fig 1A) was measured (Leica LAS version, 4.2 software) and natural log of length plotted against time (Microsoft Excel with LINEST function, Fig 3J, S3 Data). The growth curve was extrapolated back to when the trap was 10 μm long, corresponding to 1–2 cells, which we took to be the initiation stage.

### Imaging trap morphology and quadrifid glands

Propidium iodide (PI) staining was applied to fixed *U*. *gibba* traps and circinate apexes to achieve maximum depth visualisation [68]. For confocal microscopy, tissue was mounted on cavity slides with an additional gasket for added depth (Frame‐Seal Incubation Chambers, SLF0601; Bio-Rad, Hercules, CA, USA). Whole traps were imaged in sagittal orientation on either a Leica SP5-II or Zeiss LSM780 confocal microscope (excitation 514-nm laser line, detection at 580 to 660 nm; Leica). PI staining was most effective in traps <400 μm in length, above which tissue damage occurred. It was possible to get full 3D scans of PI-stained traps up to 115 μm in length. Traps larger than this were imaged to half-trap depth, ending at the dorsal midline vein.

OPT was used to visualise the full 3D shape of traps above 200 μm in length. After PI staining, traps were washed in water, embedded in 1% low melting point agarose (UltraPure LMP agarose, 16520; Invitrogen, Carlsbad, CA, USA), dehydrated overnight in methanol, and cleared in 1 part benzyl alcohol (402834; Merck):2 parts benzyl benzoate (B6630; Merck) (BABB) and prepared for OPT with a prototype scanner, as previously described [38]. UV light was used to view PI fluorescence through the Texas red (TXR) exciter filter 560/40 nm, barrier filter 610 LP. White light was used for a transmission OPT channel.

Live traps from transgenic plants containing fluorescence markers (see constructs section) were mounted in water in 1.2-mm cavity slides (BR475505; Merck) and imaged in sagittal orientation with a Leica SP5-II confocal microscope (10× or 20× lenses). GFP was excited at 488 nm and detected between 500–530 nm. mCherry was excited at 561 nm and detected between 575 and 630 nm. To visualise quadrifid glands, live traps containing fluorescence markers were cut in transverse plain with a razor, mounted in water in cavity slides, and imaged with a Leica SP5-II confocal or Zeiss LSM 5 Exciter (Zeiss, Cambourne, UK) microscope.

To view quadrifid gland pattern in three dimensions with OPT, traps were stained overnight in 10 ml water with 20 μl of 2.5% w/v Toluidine Blue (198161; Merck). Stained traps were washed in water, embedded in 1% low melting point agarose (UltraPure LMP agarose, 16520; Invitrogen), and OPT-scanned in water, with no fixation or clearing, using white light transmission OPT on a prototype OPT scanner (Lee and colleagues [38]).

### Image visualisation and quantification

Confocal image Z-stacks were converted to .png format with Bioformats converter (http://www.openmicroscopy.org/bio-formats/) or FIJI (https://fiji.sc/). OPT images were aligned using NRecon (Version 551 1.6.3.3, copyright SkyScan, 2010). VolViewer visualisation and measurement software (http://cmpdartsvr3.cmp.uea.ac.uk/wiki/BanghamLab/index.php/VolViewer) was used to view, clip, and combine GFP and mCherry fluorescence channels from confocal microscopy and fluorescence and transmission channels from OPT-scanning. Virtual dissection by cropping surrounding tissue was necessary for young traps enclosed in the circinate apex or traps with overlying stolon or leaf tissues. Measurements were made, and points were manually placed to count cells and define quadrifid gland vertices. VolViewer measurements are accurate to approximately ±5%.

The polarity of quadrifid glands was determined semiautomatically. VolViewer software was used to manually place five points on each gland, one on each arm tip and one in the centre (Fig 12A–12C). Vertices for each gland were stored as separate VolViewer objects in an MSR text file. A MATLAB script (*quadrifidScript*.*m*) calculated gland axial orientation and polarity from VolViewer vertex information. The axis was determined by calculating angles generated by each pair of arm vertices with the central vertex (6 unique pairs). The smallest and second smallest angles were found to reliably identify pairs of arms closest together and defined the axial orientation of the gland. In one output of *quadrifidScript*.*m* (polArrow_distarms_all.msr), a polarity arrow was assigned to the pair of arms with greatest distance between tips (DistArms), with a threshold of 2 μm. Quadrifid glands with a difference in distance between the two sets of arms below the threshold were shown with a line. A histogram displaying the difference between arm pair distances was also produced. A tailored version of VolViewer (Jerome Avondo) was used to display .msr files output from *quadrifidScript*.*m* (Fig 12E and 12F, Fig 12H and 12I, Fig 12K and 12L). Lloyd [13] observed shorter arms of quadrifid glands orient toward the mouth. We tested this by measuring the sum of arm length (SumArms) and allocating arrow heads toward the shortest arms (polArrow_sumarms.msr). Alone, this measure did not improve on DistArms. However, SumArms could be used as a further criterion of support to identify cases identified by DistArms in which SumArms gave the same polarity assignment (polArrow_distAndSum_all.msr). This gave a more consistent polarity field than DistArms alone (S8 Fig, S8 Data). Software used may be found here: DOI 10.6084/m9.figshare.8966153, Figs 12 and S8.7z archive.

### *U*. *gibba* development

To allocate traps to developmental stages according to time (DAI or Hours After Initiation [HAI]), confocal and OPT data sets were clipped to the centre in the sagittal plane in VolViewer, and length was measured from the dorsal lip landmark (Fig 1C) to the furthest point at the rear of the trap (Fig 1A). This length was used to place traps on the mean growth rate trend line (Fig 3J) with a Microsoft Excel histogram macro (S4 Data). To account for shrinkage on dehydration and clearing (5.78% ± 0.45 [*n* = 6], S9 Data), trap length of fixed traps was increased by 5.78% and DAI or HAI calculated as above.

Trap circumferences and regions (Fig 3, S3 Data) and trap thickness (S2 Fig, S10 Data) were manually measured with VolViewer, and cells were counted (Fig 8A–8F, S5 Data) on clipped planes: sagittal plane was clipped to dorsal midline vein, frontal plane was clipped to front of stalk in the tallest central trap region, and transverse plane was clipped to the trap centre between mouth threshold and trap door. Where half-traps were imaged, circumference measurements and cell counts for transverse and frontal planes were doubled (Fig 3K–3L and Fig 8G–8L).

Data were plotted as histograms in Microsoft Excel with LINEST function for trend line growth rate and standard deviation calculations (S3 Data, S5 Data).

To view trap shape circumferences in each plane, images from VolViewer were cropped in Adobe Photoshop and ellipses fitted and combined with Adobe Illustrator. For mature traps, OPT images had ellipses fitted in transverse and frontal views (Fig 2E and 2H, Fig 2F and 2I). Shape was traced for the sagittal circumference (Fig 2G and 2J) (mean 899 μm long, 10.4 DAI, *n* = 6). For young traps, ellipses were applied to confocal trap images (mean 55 μm long, 4 DAI, *n* = 7) (Fig 2O–2T, S1 Data, and S2 Data).

### Impact of triggering on mature trap shape

Mature culture-grown traps imaged using OPT did not exhibit the concave wall shape seen in primed, glasshouse-grown traps in water (Fig 1B, Fig 2E and 2F, S1 Fig). To determine how growth of traps cultured in vitro (in liquid 1B culture medium grown in a controlled environment room at 25°C, with 16-hour light and 8-hour dark cycles) and staining/clearing for OPT influenced shape, traps were mechanically triggered and imaged. *U*. *gibba* traps grown in a glasshouse (22°C) in water were imaged from above under bright-field light (Leica M205C stereomicroscope with Leica DFC495 camera) before and after mechanical triggering with fine forceps (S1A Fig, S1B Fig). Different live-water–grown traps, with and without triggering, were embedded in 1% low melting point agarose (UltraPure LMP agarose, 16520; Invitrogen), and OPT-imaged in water on a prototype OPT scanner [38] (S1E Fig, S1J Fig). To explore whether dehydration and clearing for OPT caused triggering, traps grown in water and in vitro in liquid 1B medium were first OPT-imaged in water, then dehydrated overnight in methanol and cleared in BABB (Merck) and OPT-imaged again (S1K–S1V Fig). Dehydration and clearing acted to trigger the trap, resulting in rounder shapes (S1O–S1P Fig, S1U–S1V Fig). Traps grown in water were larger than those grown in liquid 1B medium and showed greater shape change on triggering. To determine whether culture-grown traps could regain the primed shape, traps were imaged under bright-field light on a Leica M205C stereomicroscope with Leica DFC495 camera from above before and 24 h after transfer from liquid 1B culture medium to water, demonstrating recovery of the primed shape of water-grown traps (S1C–S1D Fig, S9 Data).

### Constructs

The 35S::loxP-RFP-loxP-GFP-HSP18::CRE-35S::Kan (EC71194) and 35S::GFP-DR5::RFP-35S::Kan (EC71257—note that we only imaged the GFP signal, not the DR5-driven RFP) constructs were created by Golden Gate cloning in the vector pAGM4723 (#48015; Addgene, Watertown, MA, USA) as previously described [69]. See S1 Methods (EC71194) and S2 Methods (EC71257) for final construct sequences and S1 Table for Golden Gate module details.

### *U*. *gibba* transformation

Cultured *U*. *gibba* stolons were divided into pieces 2–3 cm long and placed on solid media Ug 0 (4.4 g/l Murashige and Skoog Basal Medium with Vitamins (MS*, M519; PhytoTechnology Laboratories, Lenexa, KS, USA), 25 mg/l sucrose, MES hydrate, 2.5 g/l Gelrite agar (Gelzan CM, G1910; Merck) [pH 5.8] in 90 × 20 mm round Petri dishes. When the plate was covered with growth, short pieces (1–1.5 cm) were cut and placed in 5–6 clusters on Ug1 media (4.4 g/l MS*, 25 mg/l sucrose, 2.5 g/l Gelrite, 1 mg/l 6-BA [6-Benzylaminopurine (B3408; Merck)], 0.5 mg/l 1-Naphthaleneacetic acid [NAA, N0640; Merck] [pH 5.8]) in a Petri dish for 3–6 weeks. A single *Agrobacterium tumefaciens* colony (strain GV3101) containing the construct of interest (see above) was inoculated in 5 ml liquid LB medium with 50 mg/l Kanamycin (Km, 60615; Merck) and 100 mg/l Rifampicin (Rif, R3501; Merck) at 28°C for 16–24 hours. 2 ml of this culture was pelleted and resuspended in Ug2 liquid medium (4.4 g/l MS*, 25 mg/l sucrose, 1 mg/l 6-BA, 0.5 mg/NAA, pH5.8, 20 mg/l Acetosyringone [AC, D134406; Merck]) in a sterile 50 ml centrifuge tube to a resulting optical density of 0.1 to 0.2 (approximately 40 ml). 3–4 clusters of *U*. *gibba* stolon were cut to 1–1.5 cm lengths and added to the *Agrobacterium* resuspension and vacuum-infiltrated for 1 minute, then incubated for 5 minutes at room temperature. Infected explants were blotted on sterile Whatman paper to remove excess *Agrobacterium* before placing them in small clusters on solid Ug2-1 medium (Ug2 liquid media with 2.5 g/l Gelrite) for cocultivation for 3 days at 23°C in the dark. Explant clusters were then transferred to Ug3 media (4.4 g/l MS*, 25 mg/l sucrose, 2.5 g/l Gelrite, 1 mg/l 6-BA, 0.5 mg/NAA [pH 5.8], 250mg/l Cefotaxime [CEF, C7039; Merck]) for 2 weeks, then transferred to Ug4 medium (4.4 g/l MS*, 25 mg/l sucrose, 2.5 g/l Gelrite, 1 mg/l 6-BA, 0.5 mg/NAA [pH 5.8], 250 mg/l CEF, approximately 150mg/l G418 [A1720; Merck]), changing to new media every 2 to 3 weeks. After a month on Ug4 selection, most explants were dead, and only transformed explants survived and elongated quickly. Regions showing both antibiotic resistance and GFP fluorescence were transferred to Ug3 media and checked again by fluorescence microscopy.

35S::loxRFPloxGFP-HSP18::CRE-35S::Kan (EC71194) HS-inducible plants were screened for GFP and mCherry fluorescence on a Leica DM6000 fluorescence microscope, and any lines with GFP fluorescence before heat shocking were discarded. Transformed plants were confirmed to be single-copy by iDnaGENETICS, Norwich, UK. Samples were analysed by qPCR using a multiplexed taqMan reaction assaying for *NPT2* and the 35S promoter. Cofactor of nitrate reductase and xanthine dehydrogenase (*CNX3*, Scf00029.g3638.t1) was used as a single-copy control. *CNX3* was reported to be single-copy in *U*. *gibba* (Ibarra-Laclette and colleagues [22]), and this was confirmed by BLAST analysis within genome assemblies of the Bergh Apton accession used in this study [70].

### Clonal analysis

Growing stolon tips of plants, 2–3 cm in length, were collected for HS treatment and placed in six-well plates (657160; Greiner Bio-One LTD, Stonehouse, UK), each well containing 5 ml 1B media (see Plant material and specimen preparation section above) and 4–6 growing tips. Plates were sealed (Micropore tape) and floated in a 45°C water bath for 6–8 minutes. HS tissue was left to grow under standard in vitro conditions for 4 days. Traps 772–1090 μm in length (10 to 11 DAI) showing GFP clones were selected by visualising them under a fluorescence microscope (Leica Fluo III stereo or Leica DM6000). These traps were imaged in sagittal view and rotated under the coverslip or cut with a razor to allow imaging of GFP clones at multiple angles with a Leica SP5 II confocal microscope (Fig 11M–11P).

Clone anisotropy was calculated by dividing clone length along its longest (major) axis by width along the perpendicular (minor) axis (Fig 11U, S7 Data). Cell-number anisotropy was calculated by manually counting cells along the major and minor clone axes and taking the ratio (VolViewer) (Fig 11V, S7 Data). Cell-shape anisotropy is clone anisotropy/cell-number anisotropy (Fig 11W, S7 Data). To view clone shape and orientation from multiple traps together, clone images from VolViewer were cropped in Adobe Photoshop and ellipses and major axes fitted and placed in their approximate location on a cartoon trap outline with Adobe Illustrator (Fig 11Q and 11T, S17 Data).

### Segmentation

Owing to the distinct qualities of images of PI-stained fixed traps and GFP-expressing live traps, two different processing pipelines were applied to extract cellular information from confocal image stacks (Figs 9 and 10).

For GFP-expressing live traps, a Hessian-based membrane enhancing filter [71] was first used to enhance the definition of the outer surface of the trap. Thresholding and morphological operations, followed by a level-set segmentation [72], were used to locate the surfaces of the outermost layer of cells. Triangulated bladder surfaces were extracted from these binary masks using a basic 3D surface net [73], positioning vertices at the voxel centroids. Bilaplacian smoothing was used to give a smoother surface. Considering the signal intensity along a line segment normal to each vertex, vertices were moved to the point with maximum signal intensity in order to better capture the outermost surface of the outer layer of cells. Repeated rounds of bilaplacian smoothing and surface subdivision (splitting each triangular face into four) were used to generate the final refined surface. Stack signal fluorescence was projected onto each vertex of the triangulated surface [74]. Regions occupied by each cell were identified using the Surface Segmentation Potts Model (SurfaceSPM), which extends the method (Segmentation Potts Model [SPM], details to be published elsewhere) to surface image data on triangulated surfaces. The SurfaceSPM is a stochastic procedure, and combining five segmentation runs for each trap with differently-seeded random number generators yielded more accurate segmentations. The SurfaceSPM procedure sometimes generates disconnected cell labels, so labelled regions were divided into connected components, and very small (<0.05 mean label area) components were removed.

Surfaces were clipped using a manually specified polygonal region. Cells touching the edge of the clipped surface or above some size threshold (5 times the mean cell area) removed. For the purposes of cell-number quantification, gland cells were identified as cells with areas smaller than 0.25 times the mean cell area.

Early traps, imaged using PI staining, were hidden within a tight spiral structure. As discussed before, VolViewer was used to identify the region occupied by the trap. Segmentation methods (based on SPM) were used to label the regions occupied by each cell in three dimensions. Labelled regions in the exterior to the trap and within stolons were manually identified and removed.

Through binary erosion, cells protruding from the trap surface were eliminated. Morphological operations and surface nets were used to extract a triangulated surface approximating the outer surface of the trap. This surface was smoothed and translated a small distance inwards along the surface normal and underwent further refinement and smoothing.

Optimized segmentation results, either from the segmentation step of the MARS pipeline [71] (reimplemented by timagetk http://gitlab.inria.fr/mosaic/timagetk) or from the 3D SPM, generated a labelled 3D stack. Triangles of the extracted surface were assigned labels according to the label of the voxel containing their centroid. Following this step, surface label data was processed in the same manner as for the GFP stacks.

Cell areas were calculated as the sum of the areas of the triangles occupied by each cell. Cell anisotropies were calculated using the second moment of area, M, which is a matrix with entries

$$M_{ij}=\int \;(x_{i}-x_{i}^{c})(x_{j}-x_{j}^{c})d\;A,$$

where the integral is over all triangles with the label of the cell and *x*^*c*^ is the centroid of the cell. The eigenvalues of this symmetric matrix were calculated, and the anisotropy measure, *a*, is given by

$$r=\sqrt{\frac{\lambda_{1}}{\lambda_{2}}},a=\frac{r-1}{r+1}.$$

Segmentation software may be found here: https://github.com/jfozard/gibba_analysis.

### Modelling framework

All models were produced using the GPT framework [40] with GFtbox software, a MATLAB application from http://cmpdartsvr3.cmp.uea.ac.uk/wiki/BanghamLab/index.php/Software.

Models used to generate each figure can be downloaded from http://cmpdartsvr3.cmp.uea.ac.uk/wiki/BanghamLab/index.php/Software or **https://doi.org/10.6084/m9.figshare.8966153.v1**, Models.7z archive.

### Model descriptions

**Fig 4A–4C, Fig 4E and 4F: Isotropic growth promoted by MID at midline.** Prior to growth, factor MID was generated along the midline of the initial canvas and allowed to diffuse from this source with a fixed decay rate. Diffusion was inactivated prior to growth, after which MID concentrations were fixed to the canvas and deformed with it. There are three parameters in the model (*b*_*planar*_, *p*_*mid*_, *b*_*thickness*_) constrained by linear growth rate measurements. The KRN rate equations are as follows: *K*_*par*_ = *b*_*planar*_ · *pro* (*p*_*mid*_, *i*_*mid*_); *K*_*per*_ = *K*_*par*_; and *K*_*nor*_ = *b*_*thickness*_, where *b*_*planar*_ = 0.0145 is the basic specified growth rate, constrained in accordance with experimental data to give a specified areal planar growth rate of 0.029 h^−1^ (2*b*_*planar*_). *p*_*mid*_ = 0.165 is the promotion coefficient of MID on growth such that *K*_*per*_ + *K*_*par*_ = 0.033 h^−1^. *b*_*thickness*_ = 0.005 h^−1^ is the specified growth rate in thickness of the spherical sheet and is set to an experimentally observed average. *i*_*mid*_ is level of MID factor at each location in the canvas (established during the initial set up). *pro*(*z*, *i*_*y*_) denotes multiply by (1 + *zi*_*y*_).

Because *K*_*par*_ = *K*_*per*_, specified growth is isotropic, and there is only areal conflict.

**Fig 4G–4J: Isotropic growth as in Fig 4A–4E with growth inhibited by STK.** Factor STK was expressed at the “South pole” of the canvas. There are four parameters in the model (*b*_*planar*_, *p*_*mid*_, *h*_*stk*_, *b*_*thickness*_) constrained by linear growth rate measurements. KRN rate equations are as follows: *K*_*par*_ = *b*_*planar*_ · *pro*(*p*_*mid*_, *i*_*mid*_) · inh(*h*_*stk*_, *i*_*stk*_); *K*_*per*_ = *K*_*par*_; *K*_*nor*_ = *b*_*thickness*_, where *h*_*stk*_ = 1.4 is the inhibition coefficient of STK on growth such that resultant areal strain rate of the stalk region is approximately 0.015 h^−1^. *i*_*stk*_ is level of STK factor, and inh(*z*, *i*_*y*_) denotes multiply by 1/(1 + *zi*_*y*_). Values of *b*_*planar*_, *p*_*mid*_, *b*_*thickness*_ are as in the model for Fig 4A–4C and Fig 4E.

**Fig 4K–4N: Isotropic growth as in Fig 4G–4J with growth promoted by VEN.** Factor VEN was expressed in a ventral subdomain of MID. There are five parameters in the model (*b*_*planar*_, *p*_*mid*_, *h*_*stk*_, *p*_*ven*_, *b*_*thickness*_) constrained by linear growth rate measurements. KRN rate equations are as follows: *K*_*par*_ = *b*_*planar*_ · *pro*(*p*_*mid*_, *i*_*mid*_) · *inh* (*h*_*stk*_, *i*_*stk*_) · *pro* (*p*_*ven*_, *i*_*ven*_); *K*_*per*_ = *K*_*par*_; and *K*_*nor*_ = *b*_*thickness*_, where *p*_*ven*_ = 0.2 is the promotion coefficient of VEN on growth such that resultant strain rate along the ventral midline of approximately 0.02 h^−1^, matching observed measurements. *i*_*ven*_ is level of VEN factor. Values of *b*_*planar*_, *p*_*mid*_, *h*_*stk*_, *b*_*thickness*_ are as in the model for Fig 4G–4J.

**Fig 6A–6D: Anisotropic growth promoted by MID.** As with isotropic model (Fig 4A–4C) there are three parameters (*b*_*planar*_, *p*_*mid*_, *b*_*thickness*_) constrained by linear growth rate measurements. The KRN rate equations are: *K*_*par*_ = *b*_*planar*_ · *pro*(*p*_*mid*_, *i*_*mid*_); *K*_*par*_ = *min*(*2b*_*planar*_, *K*_*par*_); *K*_*per*_ = *2b*_*planar*_ − *K*_*par*_; and *K*_*nor*_ = *b*_*thickness*_, where *b*_*planar*_ = 0.015 is the basic specified growth rate, constrained in accordance with experimental data to give a specified areal planar growth rate of 0.03 h^−1^ (2*b*_*planar*_). *p*_*mid*_ = 0.35 is the promotion coefficient of MID on growth such that growth rate along the sagittal circumference approximately 0.0165 h^−1^ in accordance with experimental observations. *b*_*thickness*_ = 0.005 as with isotropic models. Note that *K*_*par*_ + *K*_*per*_ = 2*b*_*planar*_ = 0.03 everywhere, so there is no specified areal conflict.

**Fig 6E–6H: Anisotropic growth as in Fig 6A–6D with growth inhibition by STK.** As with isotropic model (Fig 4E–4H), there are four parameters (*b*_*planar*_, *p*_*mid*_, *h*_*stk*_, *b*_*thickness*_) constrained by linear growth rate measurements. The KRN rate equations are as follows: *K*_*par*_ = *b*_*planar*_ · *pro*(*p*_*mid*_, *i*_*mid*_) · *inh*(*h*_*stk*_, *i*_*stk*_); *K*_*par*_ = *min*(*2b*_*planar*_, *K*_*par*_); *K*_*per*_ = *2b*_*planar*_ − *K*_*par*_; and *K*_*nor*_ = *b*_*thickness*_, where *h*_*stk*_ = 1.5 is the inhibition coefficient of STK on *K*_*par*_ such that the resultant strain rate of the stalk parallel to the midline is approximately 0.0075 h^−1^. *i*_*stk*_ is level of STK factor. Values of *b*_*planar*_, *p*_*mid*_, *b*_*thickness*_ are as in the model for Fig 6A–6D.

**Fig 6I–6L: Anisotropic growth as in Fig 6E–6H with growth promoted by VEN.** As with isotropic model (Fig 4K–4N) there are five parameters (*b*_*planar*_, *p*_*mid*_, *h*_*stk*_, *p*_*ven*_, *b*_*thickness*_) constrained by linear growth rate measurements. The KRN rate equations are as follows: *K*_*par*_ = *b*_*planar*_ · *pro*(*p*_*mid*_, *i*_*mid*_) · *inh*(*h*_*stk*_, *i*_*stk*_) · *pro*(*p*_*ven*_, *i*_*ven*_); *K*_*par*_ = *min*(*2b*_*planar*_, *K*_*par*_); *K*_*per*_ = *2b*_*planar*_ − *K*_*par*_; *K*_*nor*_ = *b*_*thickness*_, where *p*_*ven*_ = 0.5 is the promotion coefficient of VEN on *K*_*par*_ such that the resultant RGR of the stalk parallel to the midline is approximately 0.02 h^−1^. *i*_*ven*_ is level of VEN factor. Values of *b*_*planar*_, *p*_*mid*_, *h*_*stk*_, *b*_*thickness*_ are as in the model for Fig 6E–6H.

**Fig 6M–6P: Integrated model with both areal and directional conflict.** There are seven parameters in the KRN (*b*_*planar*_, *p*_*mid*_, *h*_*stk*_, *p*_*ven*_, *b*_*thickness*_, *h*_*ven*_, *t*_*ven*_), the first five of which are constrained by linear growth rate measurements (as in Fig 4K–4N and Fig 5I–5L). The parameters *h*_*ven*_ and *t*_*ven*_ were adjusted to give a pattern of cell areas and anisotropies similar to those seen in Fig 8C and 8D and Fig 9C and 9D. The KRN rate equations are as follows: *K*_*par*_ = *b*_*planar*_ · *pro*(*p*_*mid*_, *i*_*mid*_) · *inh*(*h*_*stk*_, *i*_*stk*_) · *pro*(*p*_*ven*_, *i*_*ven*_); *K*_*per*_ = *b*_*planar*_ · *inh*(*h*_*stk*_, *i*_*stk*_) · *inh*(*h*_*ven*_, *i*_*ven*_ > *t*_*ven*_); and *K*_*nor*_ = *b*_*thickness*_, where *i*_*ven*_ > *t*_*ven*_ sets *i*_*ven*_ to a value of 1 where it exceeds the threshold value *t*_*ven*_, allowing the domain of VEN action to be widened, and where *b*_*planar*_ = 0.015, *p*_*mid*_ = 0.35, *h*_*stk*_ = 1.5, *p*_*ven*_ = 0.5, *h*_*ven*_ = 0.8 is the inhibition coefficient of VEN on *K*_*per*_, *t*_*ven*_ = 0.01, and *b*_*thickness*_ = 0.005.

**Fig 7: Model of different trap types.** The models are the same as for Fig 6 except for the following parameter variations. Terminal type: *p*_*mid*_ = 0.05, *p*_*ven*_ = 1.25. Basal type: *p*_*mid*_ = 0.45, *p*_*ven*_ = 0.1.

**S3 Fig: Variation in trap thickness.** The model is the same as for Fig 6 except that growth in thickness is promoted in the STK and VEN regions: *K*_*nor*_ = *b*_*thickness*_ · *pro*(*p*_*th*_, *i*_*stk*_) · *pro*(*p*_*th*_, *i*_*ven*_ > *t*_*ven*_), where *p*_*th*_ = 0.5.

## Supporting information

S1 Fig Impact of triggering on mature trap shape. (A–B) Shape change after manually triggering a trap. (A) Glasshouse water-grown primed trap before triggering, top view. (B) Trap shown in (A) in relaxed state after triggering with forceps. These results show that in the primed state, the traps had straight or concave side walls, whereas in the relaxed state, they had a convex shape. (C–D) Resetting shape of in vitro-grown trap by transferring it to water. (C) Primed trap grown in vitro. (D) Same trap as shown in (C) after 24 H in water, giving a more concave shape. These results indicate that the trap is in the relaxed state when grown in vitro and acquires the primed state when transferred to water. (E–G) Volume view of primed glasshouse-grown trap OPT-scanned in water. (F) Clipped frontal view of primed trap shown in (E). (G) Clipped transverse view of trap shown in (E). Note concave shape of side walls. (H–J) A manually triggered glasshouse-grown trap. (H) Volume view of triggered trap shown in (B) OPT-scanned in water. (I) Clipped frontal view of primed trap shown in (H). (J) Clipped transverse view of trap shown in (H). Note convex shape of side walls compared to (G). (K–M) Glasshouse-grown primed trap OPT-scanned in water. (K) Volume view, (L) frontal slice, (M) transverse slice. Note concave shape of side walls. (N–P) Same trap as shown in (K–M) dehydrated and cleared for OPT. (N) Volume view, (O) frontal slice, (P) transverse slice. Note convex shape similar to (H–J). These results show that dehydration and clearing for OPT analysis leads to the relaxed state. Water-grown traps were 27.3% ± 6.7 (*n* = 3) wider in frontal view after triggering. Dehydration and clearing for OPT caused 5.78% ± 0.45 (*n* = 6) shrinkage, S9 Data. (Q–S) In vitro-grown trap OPT-scanned in water. (Q) Volume view, (R) frontal slice, (S) transverse slice. Shape indicates it is between the fully primed and relaxed state. In vitro-grown traps were 12.7% ± 8.6 (*n* = 3) wider in the frontal view after triggering and 16.4% ± 0.6 (*n* = 6) smaller than water-grown traps, S9 Data. (T–V) Same trap as shown in (Q–S), triggered by dehydration and clearing for OPT. (T) Volume view, (U) frontal slice, (V) transverse slice. Note convex shape. This result shows that under the conditions used for imaging the traps (Figs 1–3), they were in the relaxed state. Scale bars 500 μm. Data **https://doi.org/10.6084/m9.figshare.8966153.v1**, S1 Fig.7z archive. OPT, Optical Projection Tomography; *Pr*, primed traps
(TIF)

S2 Fig Growth in trap thickness. (A) Clipped sagittal volume view of a trap illustrating dorsal midline thickness (red) and ventral midline thickness (magenta). PI-stained trap at 7.1 DAI imaged by confocal microscopy. Scale bar 100 μm. (B) Natural log of trap thickness plotted against time, S10 Data. Growth rates: Dorsal midline 0.43%h^−1^ ± 0.16 (R^2^ = 0.738856, *n* = 13), ventral midline 0.95%h^−1^ ± 0.15 (R^2^ = 0.940805, *n* = 12). Mean combined average growth rate is 0.69% h^−1^. Because dorsal midline makes up a larger proportion of the trap than the ventral midline, the growth rate in thickness of the models was set to 0.5% h^−1^. Mature traps showed 5.78% ± 0.45 shrinkage when prepared for OPT (S9 Data). To compensate for this, trap length of all fixed traps was increased by 5.78% before time (DAI) calculation. **https://doi.org/10.6084/m9.figshare.8966153.v1**, S2 Fig.7z archive. DAI, days after initiation; OPT, Optical Projection Tomography; PI, propidium iodide
(TIF)

S3 Fig Model variation in trap thickness. Result of running the integrated model with increased growth rate in thickness for the STK and VEN regions. Side view (left) and sagittal section (right). Domains colour-coded as in Fig 6O and 6P. Scale bar 500 μm. Models: http://cmpdartsvr3.cmp.uea.ac.uk/wiki/BanghamLab/index.php/Software or **https://doi.org/10.6084/m9.figshare.8966153.v1**, Models.7z archive STK, Stalk factor; VEN, Ventral factor.
(TIF)

S4 Fig Mean cell area. (A) Chart showing mean cell area (μm^2^) of lamina cells versus time (DAI), S6 Data. Mean cell area in range was calculated from segmented cells as shown in Figs 9 and 10. Small glandular cells (S6 Fig, arrowed) were excluded from the analysis. One trap was particularly large and had large cell areas. Data **https://doi.org/10.6084/m9.figshare.8966153.v1**, Figs 9, 10, S4 and S6_7z archive. DAI, days after initiation
(TIF)

S5 Fig Cellular-level area models and data. (A) Growth of areal conflict model side view coloured for cell area from starting spherical canvas at 4 DAI to resultant canvas at 10.5 DAI. (B) Areal conflict model front view. Arrow highlights larger ventral midline cells. (C) Directional conflict model, side view. (D) Directional conflict model, front view. Arrow highlights smaller ventral midline cells. Magenta line shows ventral midline; red line shows dorsal midline. Grey region shows mouth. In all images, colour scale shows cell area (μm^2^) on logarithmic scale. Data **https://doi.org/10.6084/m9.figshare.8966153.v1**, Figs 9, 10, S4 and S6_7z archive. Models: http://cmpdartsvr3.cmp.uea.ac.uk/wiki/BanghamLab/index.php/Software or **https://doi.org/10.6084/m9.figshare.8966153.v1**, Models.7z archive. DAI, days after initiation
(TIF)

S6 Fig Trap cell types. Trap side views of segmented confocal images shown in Fig 9B, coloured for cell area. Arrows highlight hemispherical gland cells that remain small. Colour scale shows cell area (μm^2^) on logarithmic scale. Data **https://doi.org/10.6084/m9.figshare.8966153.v1**, Figs 9, 10, S4 and S6_7z archive.
(TIF)

S7 Fig Cellular-level anisotropy models and data. (A) Areal conflict model side view from 4 DAI spherical canvas to 10.5 DAI resultant shape, showing cell anisotropy. Lines show orientation of the cell long axis and are shown where anisotropy exceeds 0.23. (B) Areal conflict model front view. Arrow highlights anisotropy of ventral midline cells. (C) Directional conflict model side view. (D) Directional conflict model front view. Arrow highlights anisotropy in ventral midline cells. In all images, colour scale shows cell anisotropy; cell-shape anisotropy is defined by R − 1/R + 1, where R is the ratio of the long to short axis of an ellipsoid fitted to the cell. This equation evaluates to 0 for isometric cell shape and 0.333 when the long axis is twice the short axis. Magenta line shows ventral midline; red line shows dorsal midline. Grey region shows mouth. Data **https://doi.org/10.6084/m9.figshare.8966153.v1**, Figs 9, 10, S4 and S6_7z archive and http://cmpdartsvr3.cmp.uea.ac.uk/wiki/BanghamLab/index.php/Software or **https://doi.org/10.6084/m9.figshare.8966153.v1**, Models.7z archive. DAI, days after initiation
(TIF)

S8 Fig Quadrifid gland orientation. (A–C) Quadrifid orientation side view (also shown in Fig 12E). (A) Clipped OPT sagittal view looking into trap at quadrifid glands on the left-hand wall. Arrows (magenta) orient toward greatest distance between quadrifid arms (DistArms output). Arrowheads were unassigned if distance subtraction value between arm sets was <2 μm (shown as lines). (B) DistArms above threshold (all arrows, green or black); DistArms above threshold and polarity assignment further supported by DistArmsSumArms (black arrows), DistArms below threshold (green lines). 35/37 of all arrows and 17/17 black arrows point from stalk towards mouth (S8 Data). (C) DistArms histogram plotting quadrifid number versus arm pair subtraction value (S11 Data). (D–F) Quadrifid orientation side view. (D) Clipped confocal sagittal view looking into trap, DistArms output. (E) 31/31 of all arrows and 29/29 black arrows point from stalk to mouth, three not allocated (S8 Data). (F) DistArms histogram (S12 Data). (G–I) Transverse clipped view looking into ventral half of trap, confocal scan (also shown in Fig 12H). (G) DistArms output. (H) 29/32 of all arrows and 18/18 black arrows point away from the stalk, six unallocated (S8 Data). (I) DistArms histogram (S13 Data). (J–L) Transverse clipped view looking into bottom half of trap, confocal scan. (J) DistArms output. (K) 20/24 of all arrows and 16/17 black arrows point from stalk to mouth. 3 unallocated. (L) DistArms histogram (S14 Data). (M–O) Transverse clipped view looking into top of trap. OPT scan. (M) DistArms output. (N) 39/ 43 of all arrows and 13/14 black arrows point towards the mouth. 7 unallocated (S8 Data). (O) DistArms histogram (S15 Data). (P–R) Transverse clipped view looking into top of trap, confocal scan (also shown in Fig 12K). (P) DistArms output. (Q) 27/27 of all arrows and 23/23 black arrows point towards mouth (S8 Data). (R) DistArms histogram (S16 Data). Scale bars = 100 μm. Mo = direction of mouth in trap image. St = approximate location of stalk in trap image. Data **https://doi.org/10.6084/m9.figshare.8966153.v1**, Figs 12 and S8.7z archive. Models: http://cmpdartsvr3.cmp.uea.ac.uk/wiki/BanghamLab/index.php/Software or **https://doi.org/10.6084/m9.figshare.8966153.v1**, Models.7z archive. OPT, Optical Projection Tomography.
(TIF)

S1 Movie Mature trap shape. Volume view of a PI-stained mature *U*. *gibba* trap visualised by OPT shown in Fig 2A. PI fluorescence is red, and tissue autofluorescence is green. The trap is clipped in transverse, frontal, and sagittal planes. OPT, Optical Projection Tomography; PI, propidium iodide
(MP4)

S2 Movie Shape of young trap. Volume view of young PI-stained *U*. *gibba* trap visualised by confocal microscopy shown in Fig 2K. The trap is clipped in sagittal, frontal, and transverse planes. PI, propidium iodide
(MP4)

S3 Movie Areal conflict resolution model with MID domain. Tissue-level modelling of trap development through areal conflict resolution with growth promoted by MID (red) (Fig 4A–4F). Scale bar is held constant to show increase in size from initial to resultant shape. Final shape (oblate spheroid) is rotated at the end of the movie to show MID domain in front view. MID, Midline factor.
(AVI)

S4 Movie Areal conflict resolution model with MID and STK domains. Tissue-level modelling of trap development through areal conflict resolution with growth promoted by MID (red) and inhibited by STK (green) (Fig 4G–4J). Scale bar is held constant to show increase in size from initial to resultant shape. Note indentation at the base of the trap where STK inhibits growth. Final shape is rotated at the end of the movie to show MID domain in front view. MID, Midline factor; STK, Stalk factor.
(AVI)

S5 Movie Areal conflict resolution model with MID, STK, and VEN domains. Tissue-level modelling of trap development through areal conflict resolution with growth promoted by MID (red) and VEN (magenta) and inhibited by STK (green) (Fig 4K–4N). Scale bar is held constant to show increase in size from initial to resultant shape. Final shape is rotated at the end of the movie to show MID and VEN domains in front view. Note the bulge of the ventral midline region (magenta). MID, Midline factor; STK, Stalk factor; VEN, Ventral factor.
(AVI)

S6 Movie Directional conflict resolution model with specified anisotropy promoted by MID. Tissue-level modelling of trap development through directional conflict resolution. Specified anisotropy, defined as (*K*_*par*_ − *K*_*per*_)/(*K*_*par*_ + *K*_*per*_) promoted by MID (red) (Fig 6A–6D). Scale bar is held constant to show increase in size from initial to resultant shape. Final shape (oblate spheroid) is rotated at the end of the movie to show MID domain and −ORG (cyan) in front view. The pointed shape at the base of the trap is where +ORG is located. Polarity runs from +ORG to −ORG. MID, Midline factor; −ORG, minus-organiser; +ORG, plus-organiser.
(AVI)

S7 Movie Directional conflict resolution with specified anisotropy promoted by MID and inhibited by STK. Tissue-level modelling of trap development through directional conflict resolution. Specified anisotropy, defined as (*K*_*par*_ − *K*_*per*_)/(*K*_*par*_ + *K*_*per*_) is positive in MID domain (red) and negative in STK domain (green) (Fig 6E–6H). Scale bar is held constant to show increase in size from initial to resultant shape. Final shape is rotated at the end of the movie to show MID domain and −ORG (cyan) in front view. Note indented shape at the base of the trap where *K*_*par*_ is inhibited by STK. MID, Midline factor; STK, Stalk factor; −ORG, minus-organiser.
(AVI)

S8 Movie Directional conflict resolution with specified anisotropy promoted by VEN and MID and inhibited by STK. Tissue-level modelling of trap development through directional conflict resolution. Specified anisotropy, defined as (*K*_*par*_ − *K*_*per*_)/(*K*_*par*_ + *K*_*per*_) is positive in MID domain (red), further enhanced by VEN (magenta), and negative in STK domain (green) (Fig 6I–6L). Scale bar is held constant to show increase in size from initial to resultant shape. Final shape is rotated at the end of the movie to show MID domain and −ORG (cyan) in front view. Note the extended ventral midline region (magenta) where specified anisotropy is promoted by VEN. MID, Midline factor; STK, Stalk factor; VEN, Ventral factor; −ORG, minus-organiser.
(AVI)

S9 Movie Integrated model movie with specified anisotropy promoted by VEN and MID and inhibited by STK. Tissue-level modelling of trap development through integrated areal and directional conflict resolution (Fig 6M–6P). Scale bar is held constant to show increase in size from initial to resultant shape. Final shape is rotated at the end of the movie to show MID domain and −ORG (cyan) in front view. Note the extended ventral midline region (magenta) where specified anisotropy and growth is promoted by VEN. MID, Midline factor; STK, Stalk factor; VEN, Ventral factor; −ORG, minus-organiser.
(AVI)

S10 Movie Integrated model colour-coded for cell area. Cellular-level integrated directional and areal conflict model coloured for cell area. Grey region shows approximate location of mouth (Fig 9G, S5E Fig, S5F Fig). Scale bar is held constant to show increase in size from initial to resultant shape. Final shape is rotated at the end of the movie. Note similar size of cells in the ventral midline region compared to the rest of the mature trap.
(AVI)

S11 Movie Integrated model colour-coded for cell area (rescale). Cellular-level integrated directional and areal conflict model coloured for cell area as shown in S10 Movie but continually rescaled to show shape change normalised for size.
(AVI)

S12 Movie Areal conflict model with cell area. Cellular-level areal conflict model coloured for cell area, grey region shows approximate location of mouth (Fig 9E, S5A Fig, S5B Fig). Scale bar is held constant to show increase in size from initial to resultant shape. Final shape is rotated at the end of the movie. Note enlarged cells in the bulge of the ventral midline region of the mature trap.
(AVI)

S13 Movie Directional conflict model with cell area. Cellular-level directional conflict model coloured for cell area; grey region shows approximate location of mouth (Fig 9F, S5C Fig, S5D Fig). Scale bar is held constant to show increase in size from initial to resultant shape. Final shape is rotated at the end of the movie. Note smaller cells in the extended ventral midline region of the mature trap.
(AVI)

S14 Movie Areal conflict model with cell anisotropy. Cellular-level areal conflict model coloured for cell anisotropy (Fig 9E, S7A Fig, S7B Fig). Scale bar is held constant to show increase in size from initial to resultant shape. Final shape is rotated at the end of the movie. Note cells in the ventral midline region are mostly isotropic in the bulging ventral midline domain.
(AVI)

S15 Movie Directional conflict model with cell anisotropy. Cellular-level directional conflict model coloured for cell anisotropy (Fig 9F, S7C Fig, S7D Fig). Scale bar is held constant to show increase in size from initial to resultant shape. Final shape is rotated at the end of the movie. Note cells are anisotropic in the elongated ventral midline domain of the mature trap.
(AVI)

S16 Movie Integrated model colour-coded for cell anisotropy. Cellular-level integrated directional and areal conflict model coloured for cell anisotropy (Fig 9G, S7E Fig, S7F Fig). Scale bar is held constant to show increase in size from initial to resultant shape. Final shape is rotated at the end of the movie. Note cells are anisotropic in a wider region of the ventral midline domain of the mature trap.
(AVI)

S17 Movie Integrated model colour-coded for cell anisotropy (rescale). Cellular-level integrated directional and areal conflict model coloured for cell anisotropy as shown in S16 Movie but continually rescaled to show shape change normalised for size.
(AVI)

S18 Movie Directional conflict model with virtual clones. Virtual clones generated by directional conflict model. Clones were induced at 4 DAI. Scale bar is held constant to show increase in size from initial to resultant shape. Final shape is rotated at the end of the movie. Resultant model outputs shown are 10.5 DAI (Fig 11E–11H). DAI, days after initiation
(AVI)

S19 Movie Integrated model with virtual clones. Virtual clones generated by integrated directional and areal conflict model. Clones were induced at 4 DAI. Scale bar is held constant to show increase in size from initial to resultant shape. Final shape is rotated at the end of the movie. Resultant model outputs shown are 10.5 DAI (Fig 11I–11L), note elongated clones in ventral midline domain. DAI, days after initiation
(AVI)

S20 Movie Areal conflict model with virtual clones. Virtual clones generated by areal conflict model. Clones were induced at 4 DAI. Scale bar is held constant to show increase in size from initial to resultant shape. Final shape is rotated at the end of the movie. Resultant model outputs shown are 10.5 DAI (Fig 11A–11D). DAI, days after initiation
(AVI)

S21 Movie HS-induced clones, side view. HS-induced clones (green) imaged with a confocal microscope at 10–11 DAI (Fig 11M). DAI, days after initiation; HS, heat shock
(MP4)

S22 Movie HS-induced clones, front view. HS-induced clones (green) imaged with a confocal microscope at 10–11 DAI, additional specimen (Fig 11N). DAI, days after initiation; HS, heat shock
(MP4)

S23 Movie Assigning polarity to quadrifid glands. Point coordinates at the quadrifid gland centre and at ends of each quadrifid gland arm were placed in VolViewer. Arrowheads were assigned oriented toward the greatest distance between arms with quadrifidScript software (DistArms) (Fig 12A–12D).
(MP4)

S24 Movie Quadrifid gland polarity trap side view. OPT volume view of a *U*. *gibba* trap clipped to view quadrifid glands. Arrows flow from stalk to mouth through the side of the trap. Lines with no arrowheads were allocated when the difference in distance between arms was less than a threshold value of 2 μm (Fig 12E and 12F). OPT, Optical Projection Tomography.
(MP4)

S25 Movie Quadrifid polarity trap front view. Confocal volume view of a *U*. *gibba* trap showing quadrifid glands. Arrows flow from stalk to mouth and up sides and back of the trap. Lines with no arrowheads were allocated when the difference in distance between arms was less than a threshold value of 2 μm (Fig 12E and 12F).
(MP4)

S1 Data Trap shape outlines at mature and early stages. Ellipses shown in Fig 2H–2J and Fig 2R–2T were fitted to transverse and frontal perimeters, and sagittal view outlines were drawn in this Adobe Illustrator file.
(AI) Click here for additional data file.

S2 Data Trap diameter at mature and early stages. Trap diameter was measured in VolViewer and the mean calculated in each plane and used to scale the common line shown in trap outlines shown in Fig 2H–2J and Fig 2R–2T.
(XLSX)

S3 Data Trap growth rates. Trap growth rate source data file used to make the charts shown in Fig 3J–3L.
(XLSX)

S4 Data Developmental staging of traps. Growth rate calculated from daily imaging was used to stage traps to developmental time (DAI or HAI) with this Excel file. DAI, days after initiation; HAI, hours after initiation
(XLSX)

S5 Data Cell counts at different stages of trap development. Cell count source data file for charts shown in Fig 8G–8L.
(XLSX)

S6 Data Mean cell area. Cell area source data file for chart shown in S4 Fig.
(XLS)

S7 Data Clonal analysis. Clonal analysis source data file for charts shown in Fig 10U–10W.
(XLSX)

S8 Data Evidence for a polarity field in traps. Quadrifid orientation count data file for Fig 12 and S8 Fig.
(XLSX)

S9 Data Impact of triggering on mature trap shape. Trapping measurements and shrinkage data as shown in S1 Fig.
(XLSX)

S10 Data Growth in trap thickness. Trap thickness source data file for chart shown in S2 Fig.
(XLSX)

S11 Data Evidence for a polarity field in traps. S8C Fig source data file.
(XLSX)

S12 Data Evidence for a polarity field in traps. S8F Fig source data file.
(XLSX)

S13 Data Evidence for a polarity field in traps. S8I Fig source data file.
(XLSX)

S14 Data Evidence for a polarity field in traps. S8L Fig source data file.
(XLSX)

S15 Data Evidence for a polarity field in traps. S8O Fig source data file.
(XLSX)

S16 Data Evidence for a polarity field in traps. S8R Fig source data file.
(XLSX)

S17 Data Clonal analysis. Clonal sectors are fitted to trap outlines in this Adobe Illustrator file as shown in Fig 11Q–11T.
(AI) Click here for additional data file.

S1 Methods Full sequence for EC71194. (DOCX)

S2 Methods Full sequence for EC71257. (DOCX)

S1 Resources Key resources table. (DOCX)

S1 Table Goldengate construction. Breakdown of Goldengate parts used to create constructs used. Level 0 parts were synthesised and used to create level 0.5 and 1 constructs, and these were combined to make level 2 constructs, as described in [69].
(XLSX)

## Abbreviations

AC: Acetosyringone
BABB: 1 part benzyl alcohol: 2 parts benzyl benzoate
CaMV: Cauliflower mosaic virus
CDIV: factor conferring division competence
CEF: Cefotaxime
CNX3: cofactor of nitrate reductase and xanthine dehydrogenase
DAI: days after initiation
GFP: green fluorescent protein
GPT: growing polarised tissue
HAI: Hours After Initiation
HS: heat shock
Km: Kanamycin
KRN: Growth regulatory network
LAM: Lamina factor
LATE: Factor which increases with time
MID: Midline factor
NAA: 1-Naphthaleneacetice acid
OPT: Optical Projection Tomography
PGRAD: Factor with graded distribution that decreases from proximal to distal positions
PI: propidium iodide
POL: polariser
Rif: Rifampicin
SPM: Segmentation Potts Model
STK: Stalk factor
TXR: Texas red
VEN: Ventral factor
6-BA: 6-Benzylaminopurine
−ORG: minus-organiser
+ORG: plus-organiser
