# Supplementary figures and images for: Shaping of a three-dimensional carnivorous trap through modulation of a planar growth mechanism
Source: PLoS Biol. 2019 Oct 10;17(10):e3000427. doi: 10.1371/journal.pbio.3000427 (PMC6786542; doi:10.1371/journal.pbio.3000427)

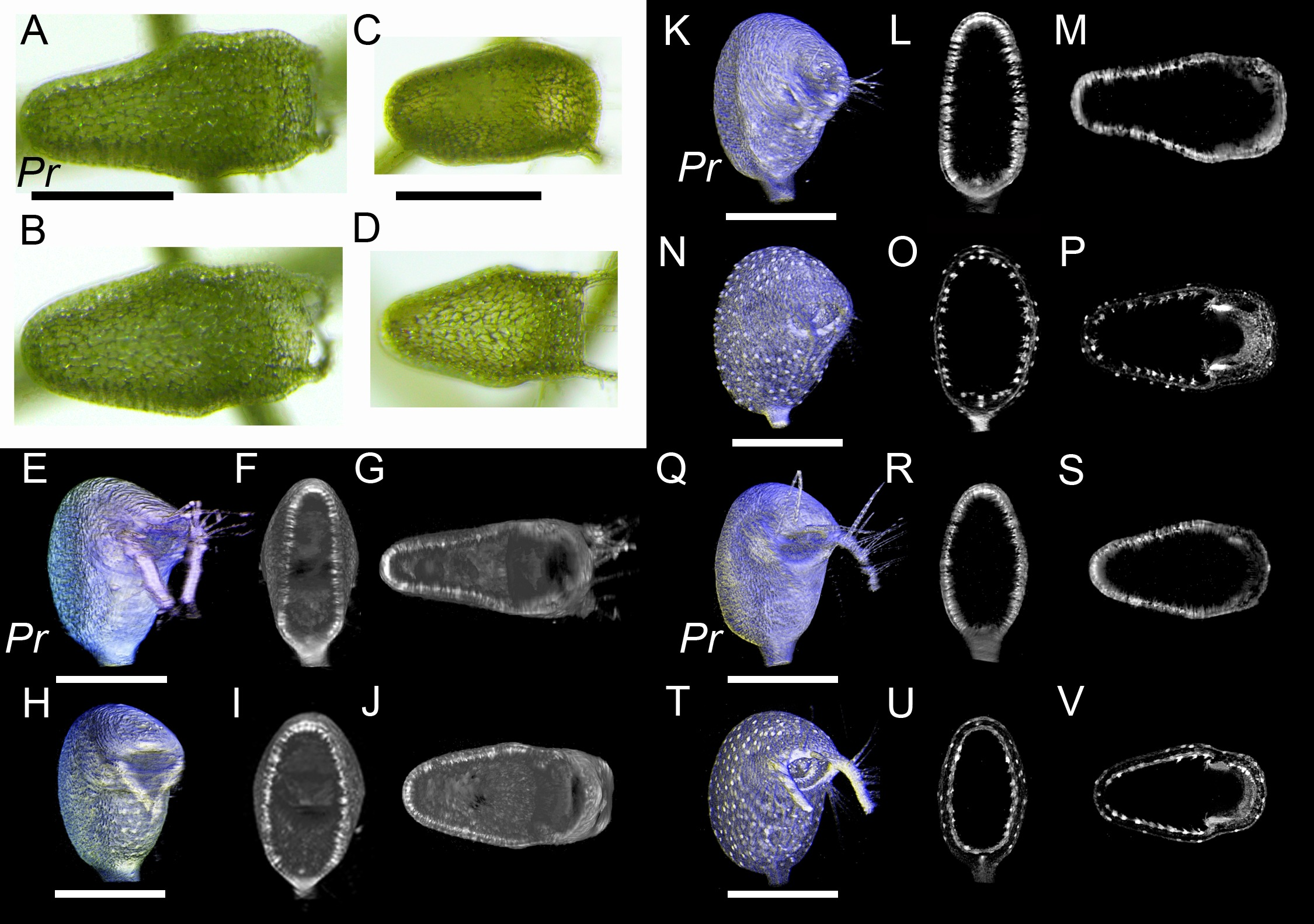

Supplement: S1 Fig — (A–B) Shape change after manually triggering a trap. (A) Glasshouse water-grown primed trap before triggering, top view. (B) Trap shown in (A) in relaxed state after triggering with forceps. These results show that in the primed state, the traps had straight or concave side walls, whereas in the relaxed state, they had a convex shape. (C–D) Resetting shape of in vitro-grown trap by transferring it to water. (C) Primed trap grown in vitro. (D) Same trap as shown in (C) after 24 H in water, giving a more concave shape. These results indicate that the trap is in the relaxed state when grown in vitro and acquires the primed state when transferred to water. (E–G) Volume view of primed glasshouse-grown trap OPT-scanned in water. (F) Clipped frontal view of primed trap shown in (E). (G) Clipped transverse view of trap shown in (E). Note concave shape of side walls. (H–J) A manually triggered glasshouse-grown trap. (H) Volume view of triggered trap shown in (B) OPT-scanned in water. (I) Clipped frontal view of primed trap shown in (H). (J) Clipped transverse view of trap shown in (H). Note convex shape of side walls compared to (G). (K–M) Glasshouse-grown primed trap OPT-scanned in water. (K) Volume view, (L) frontal slice, (M) transverse slice. Note concave shape of side walls. (N–P) Same trap as shown in (K–M) dehydrated and cleared for OPT. (N) Volume view, (O) frontal slice, (P) transverse slice. Note convex shape similar to (H–J). These results show that dehydration and clearing for OPT analysis leads to the relaxed state. Water-grown traps were 27.3% ± 6.7 (n = 3) wider in frontal view after triggering. Dehydration and clearing for OPT caused 5.78% ± 0.45 (n = 6) shrinkage, S9 Data. (Q–S) In vitro-grown trap OPT-scanned in water. (Q) Volume view, (R) frontal slice, (S) transverse slice. Shape indicates it is between the fully primed and relaxed state. In vitro-grown traps were 12.7% ± 8.6 (n = 3) wider in the frontal view after triggering and 16.4% ± 0.6 (n = 6) small [file pbio.3000427.s001.tif]

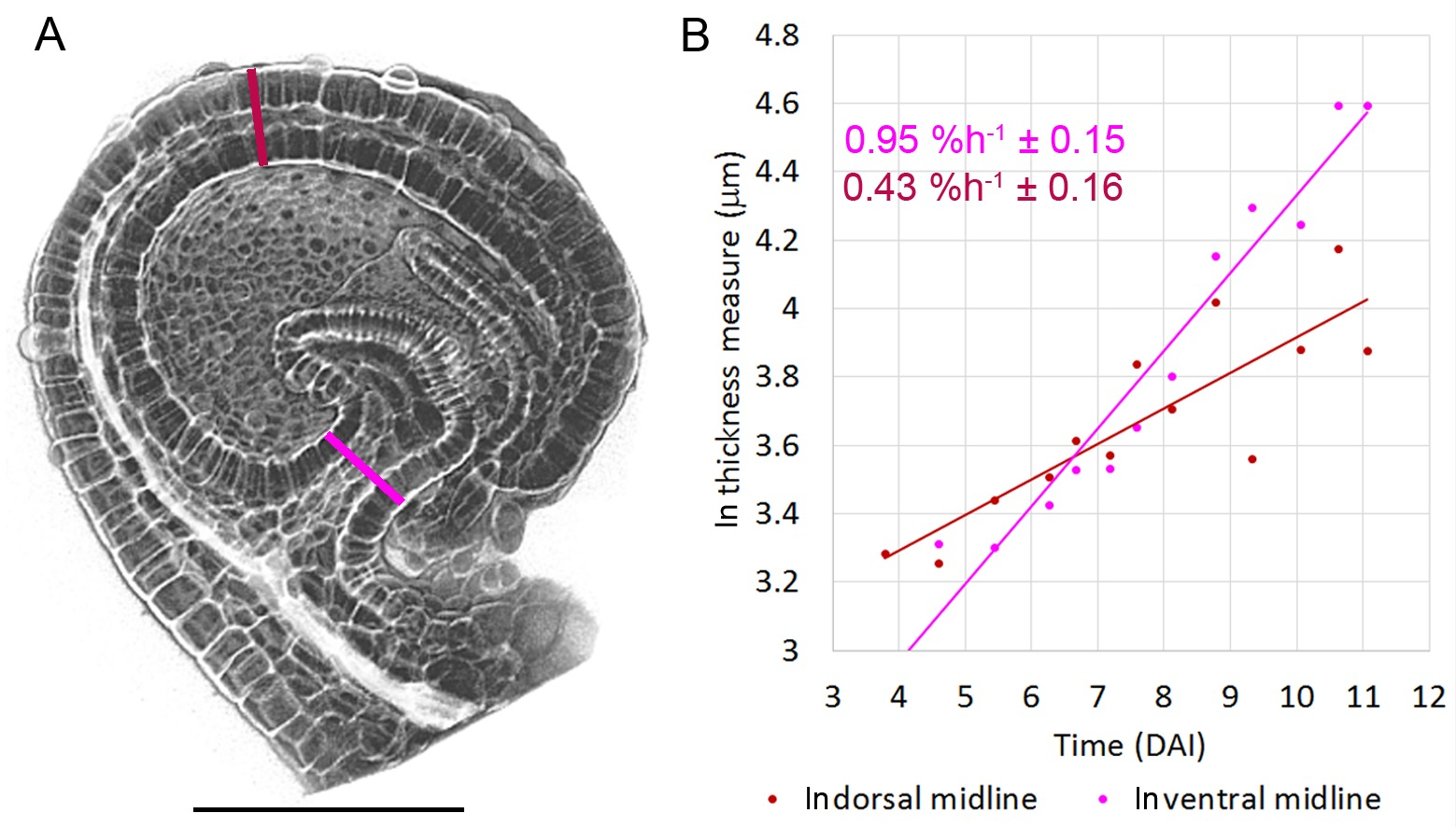

Supplement: S2 Fig — (A) Clipped sagittal volume view of a trap illustrating dorsal midline thickness (red) and ventral midline thickness (magenta). PI-stained trap at 7.1 DAI imaged by confocal microscopy. Scale bar 100 μm. (B) Natural log of trap thickness plotted against time, S10 Data. Growth rates: Dorsal midline 0.43%h−1 ± 0.16 (R2 = 0.738856, n = 13), ventral midline 0.95%h−1 ± 0.15 (R2 = 0.940805, n = 12). Mean combined average growth rate is 0.69% h−1. Because dorsal midline makes up a larger proportion of the trap than the ventral midline, the growth rate in thickness of the models was set to 0.5% h−1. Mature traps showed 5.78% ± 0.45 shrinkage when prepared for OPT (S9 Data). To compensate for this, trap length of all fixed traps was increased by 5.78% before time (DAI) calculation. https://doi.org/10.6084/m9.figshare.8966153.v1, S2 Fig.7z archive. DAI, days after initiation; OPT, Optical Projection Tomography; PI, propidium iodide (TIF) [file pbio.3000427.s002.tif]

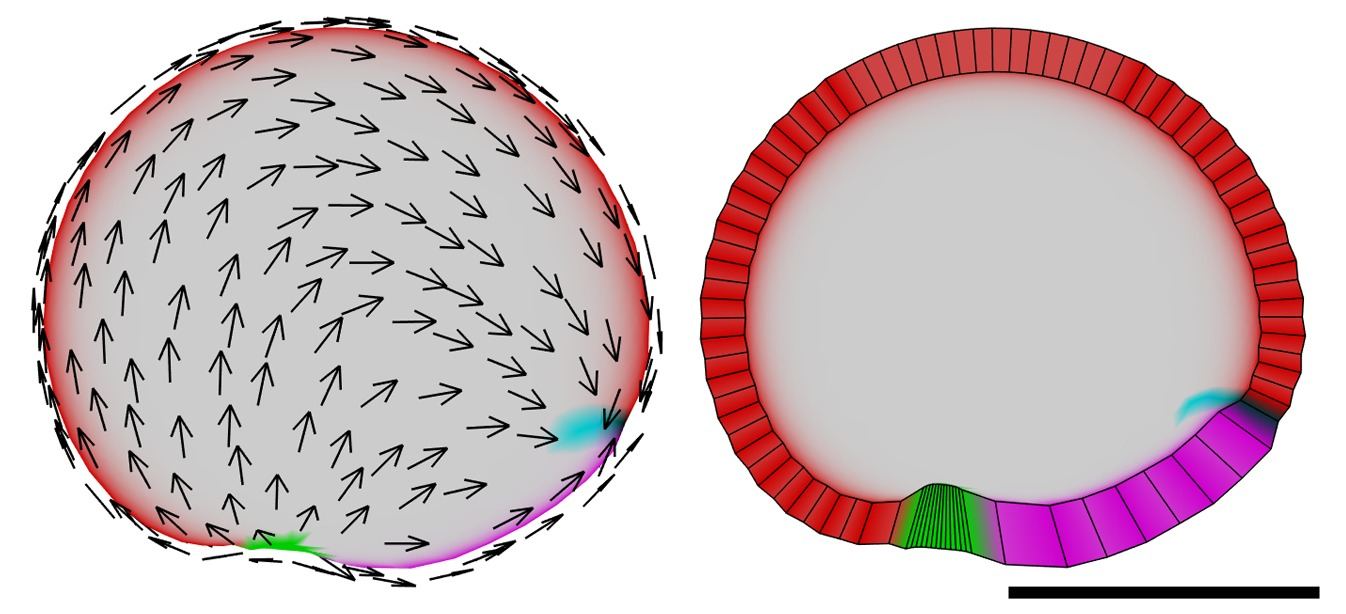

Supplement: S3 Fig — Result of running the integrated model with increased growth rate in thickness for the STK and VEN regions. Side view (left) and sagittal section (right). Domains colour-coded as in Fig 6O and 6P. Scale bar 500 μm. Models: http://cmpdartsvr3.cmp.uea.ac.uk/wiki/BanghamLab/index.php/Software or https://doi.org/10.6084/m9.figshare.8966153.v1, Models.7z archive STK, Stalk factor; VEN, Ventral factor. (TIF) [file pbio.3000427.s003.tif]

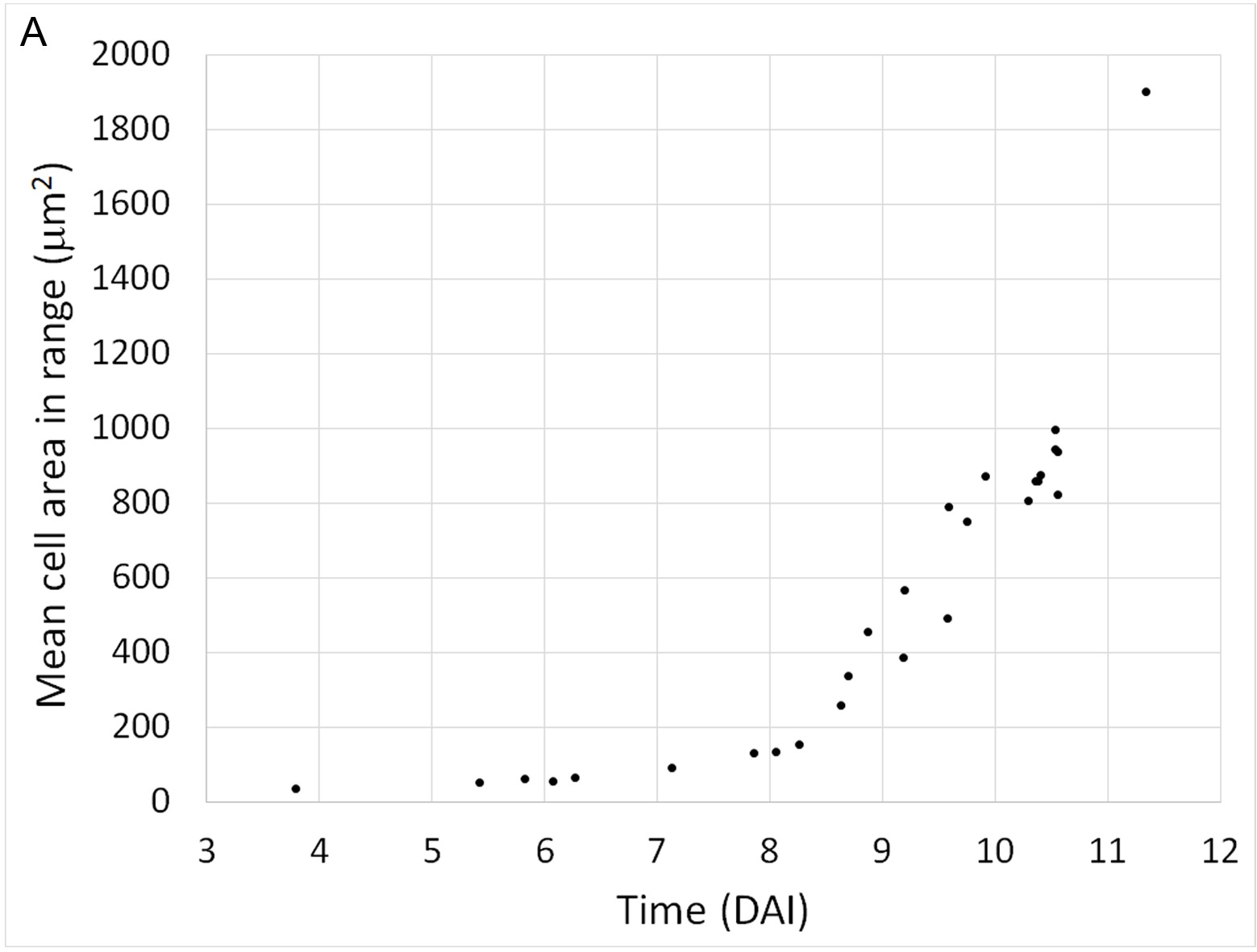

Supplement: S4 Fig — (A) Chart showing mean cell area (μm2) of lamina cells versus time (DAI), S6 Data. Mean cell area in range was calculated from segmented cells as shown in Figs 9 and 10. Small glandular cells (S6 Fig, arrowed) were excluded from the analysis. One trap was particularly large and had large cell areas. Data https://doi.org/10.6084/m9.figshare.8966153.v1, Figs 9, 10, S4 and S6_7z archive. DAI, days after initiation (TIF) [file pbio.3000427.s004.tif]

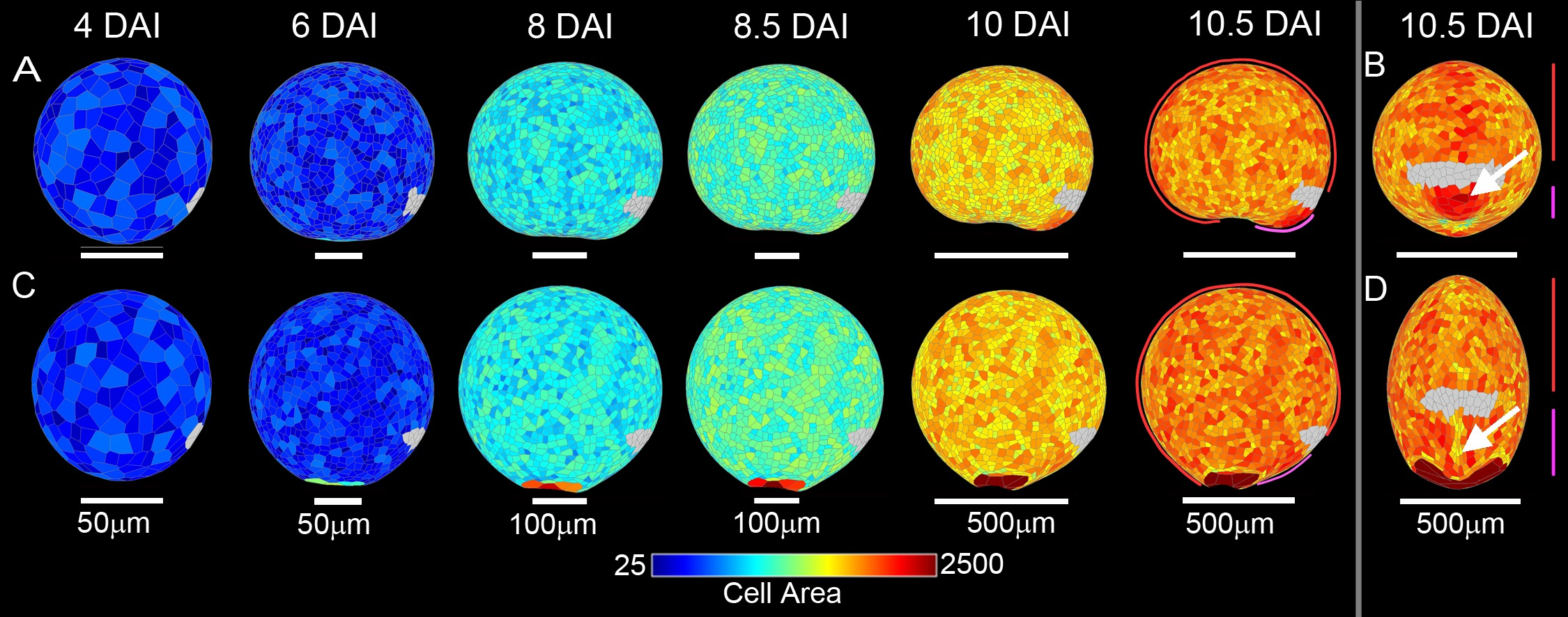

Supplement: S5 Fig — (A) Growth of areal conflict model side view coloured for cell area from starting spherical canvas at 4 DAI to resultant canvas at 10.5 DAI. (B) Areal conflict model front view. Arrow highlights larger ventral midline cells. (C) Directional conflict model, side view. (D) Directional conflict model, front view. Arrow highlights smaller ventral midline cells. Magenta line shows ventral midline; red line shows dorsal midline. Grey region shows mouth. In all images, colour scale shows cell area (μm2) on logarithmic scale. Data https://doi.org/10.6084/m9.figshare.8966153.v1, Figs 9, 10, S4 and S6_7z archive. Models: http://cmpdartsvr3.cmp.uea.ac.uk/wiki/BanghamLab/index.php/Software or https://doi.org/10.6084/m9.figshare.8966153.v1, Models.7z archive. DAI, days after initiation (TIF) [file pbio.3000427.s005.tif]

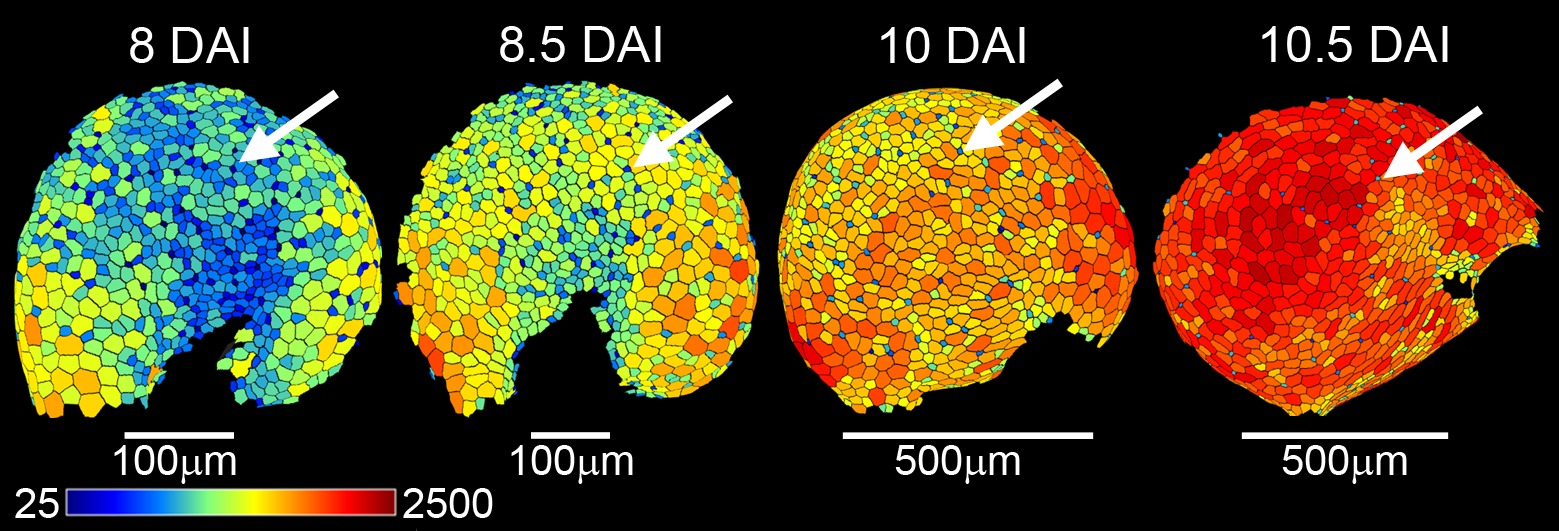

Supplement: S6 Fig — Trap side views of segmented confocal images shown in Fig 9B, coloured for cell area. Arrows highlight hemispherical gland cells that remain small. Colour scale shows cell area (μm2) on logarithmic scale. Data https://doi.org/10.6084/m9.figshare.8966153.v1, Figs 9, 10, S4 and S6_7z archive. (TIF) [file pbio.3000427.s006.tif]

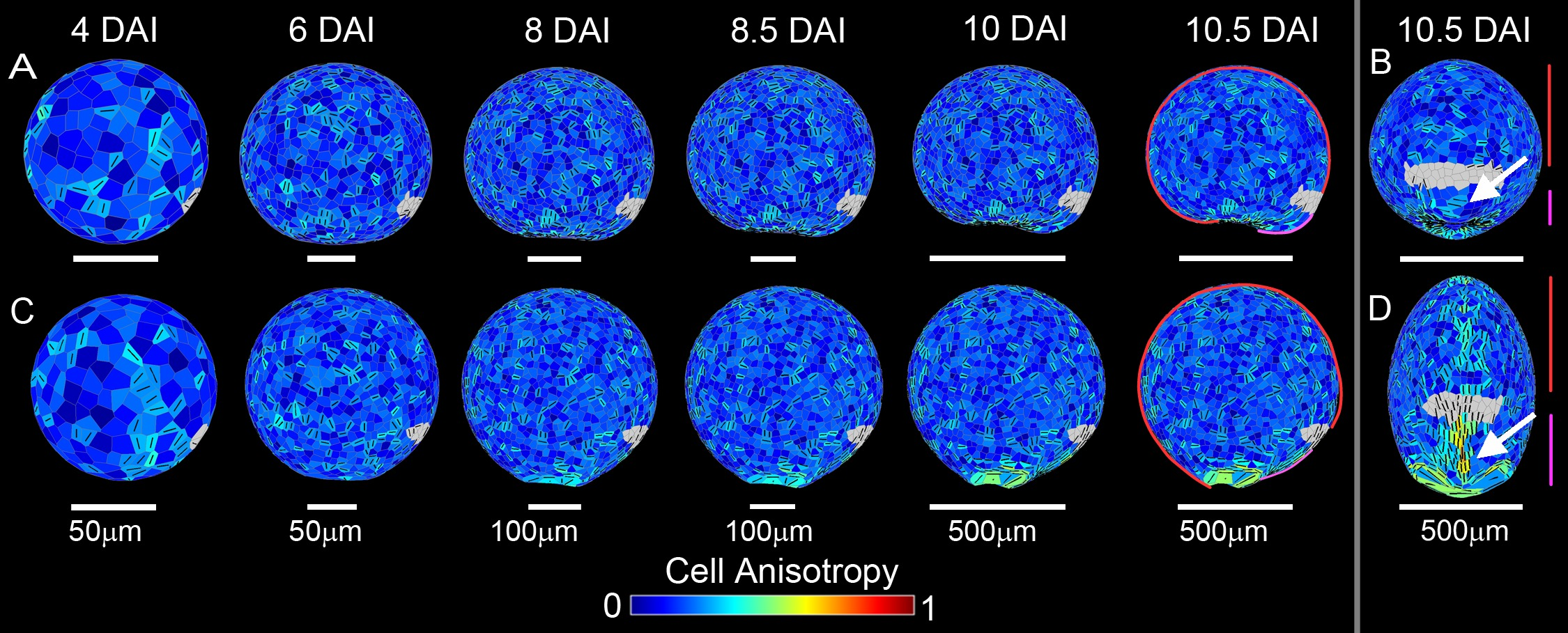

Supplement: S7 Fig — (A) Areal conflict model side view from 4 DAI spherical canvas to 10.5 DAI resultant shape, showing cell anisotropy. Lines show orientation of the cell long axis and are shown where anisotropy exceeds 0.23. (B) Areal conflict model front view. Arrow highlights anisotropy of ventral midline cells. (C) Directional conflict model side view. (D) Directional conflict model front view. Arrow highlights anisotropy in ventral midline cells. In all images, colour scale shows cell anisotropy; cell-shape anisotropy is defined by R − 1/R + 1, where R is the ratio of the long to short axis of an ellipsoid fitted to the cell. This equation evaluates to 0 for isometric cell shape and 0.333 when the long axis is twice the short axis. Magenta line shows ventral midline; red line shows dorsal midline. Grey region shows mouth. Data https://doi.org/10.6084/m9.figshare.8966153.v1, Figs 9, 10, S4 and S6_7z archive and http://cmpdartsvr3.cmp.uea.ac.uk/wiki/BanghamLab/index.php/Software or https://doi.org/10.6084/m9.figshare.8966153.v1, Models.7z archive. DAI, days after initiation (TIF) [file pbio.3000427.s007.tif]

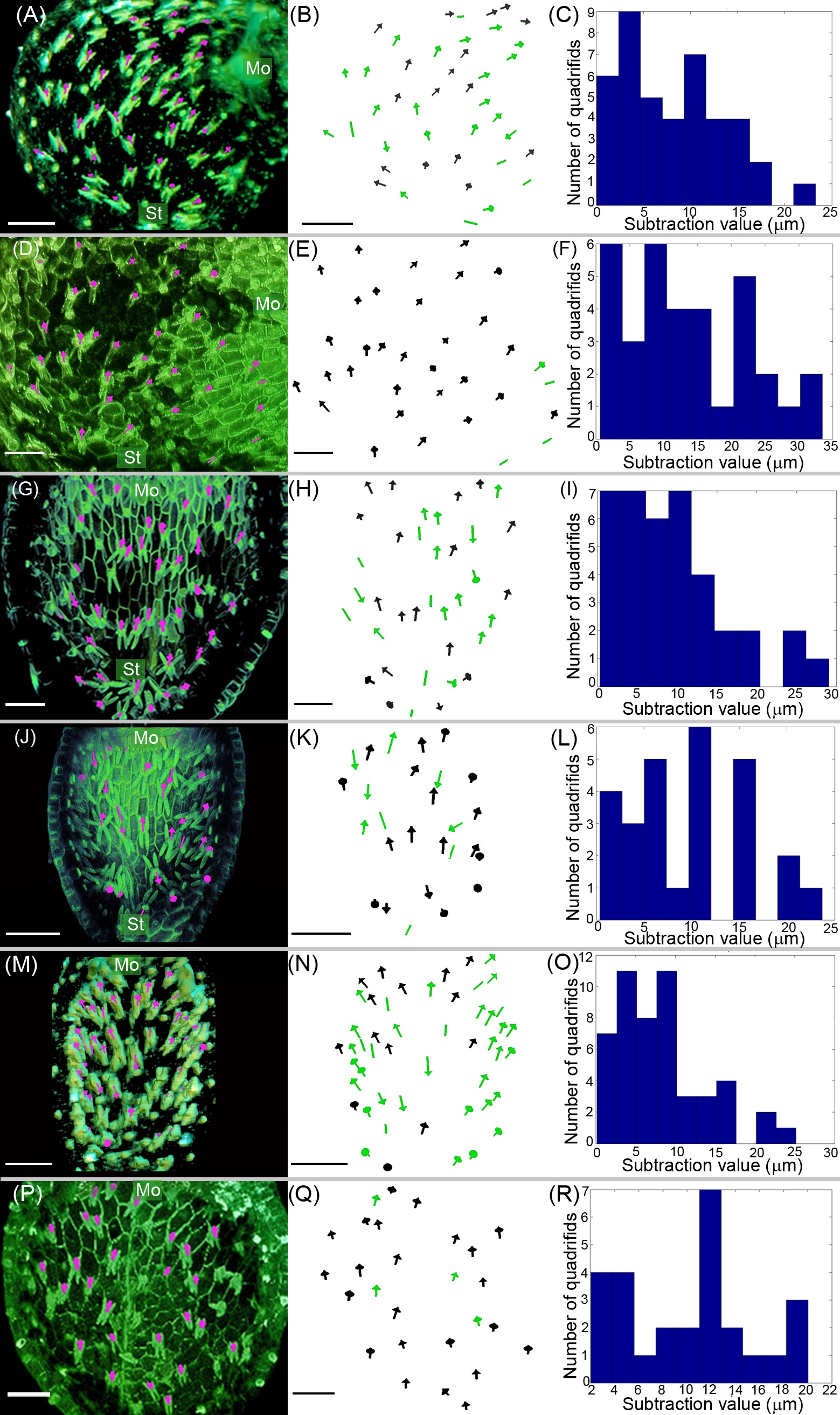

Supplement: S8 Fig — (A–C) Quadrifid orientation side view (also shown in Fig 12E). (A) Clipped OPT sagittal view looking into trap at quadrifid glands on the left-hand wall. Arrows (magenta) orient toward greatest distance between quadrifid arms (DistArms output). Arrowheads were unassigned if distance subtraction value between arm sets was <2 μm (shown as lines). (B) DistArms above threshold (all arrows, green or black); DistArms above threshold and polarity assignment further supported by DistArmsSumArms (black arrows), DistArms below threshold (green lines). 35/37 of all arrows and 17/17 black arrows point from stalk towards mouth (S8 Data). (C) DistArms histogram plotting quadrifid number versus arm pair subtraction value (S11 Data). (D–F) Quadrifid orientation side view. (D) Clipped confocal sagittal view looking into trap, DistArms output. (E) 31/31 of all arrows and 29/29 black arrows point from stalk to mouth, three not allocated (S8 Data). (F) DistArms histogram (S12 Data). (G–I) Transverse clipped view looking into ventral half of trap, confocal scan (also shown in Fig 12H). (G) DistArms output. (H) 29/32 of all arrows and 18/18 black arrows point away from the stalk, six unallocated (S8 Data). (I) DistArms histogram (S13 Data). (J–L) Transverse clipped view looking into bottom half of trap, confocal scan. (J) DistArms output. (K) 20/24 of all arrows and 16/17 black arrows point from stalk to mouth. 3 unallocated. (L) DistArms histogram (S14 Data). (M–O) Transverse clipped view looking into top of trap. OPT scan. (M) DistArms output. (N) 39/ 43 of all arrows and 13/14 black arrows point towards the mouth. 7 unallocated (S8 Data). (O) DistArms histogram (S15 Data). (P–R) Transverse clipped view looking into top of trap, confocal scan (also shown in Fig 12K). (P) DistArms output. (Q) 27/27 of all arrows and 23/23 black arrows point towards mouth (S8 Data). (R) DistArms histogram (S16 Data). Scale bars = 100 μm. Mo = direction of mouth in trap image. St = approximate location of [file pbio.3000427.s008.tif]
