# Supplementary material for: Shaping of a three-dimensional carnivorous trap through modulation of a planar growth mechanism
Source: PLoS Biol. 2019 Oct 10;17(10):e3000427. doi: 10.1371/journal.pbio.3000427 (PMC6786542; doi:10.1371/journal.pbio.3000427)
Supplement: S1 Methods — (DOCX) [file pbio.3000427.s051.docx]

## S1 Methods. Full sequence for EC71194

To produce the pL2B-KAN-Cre-p35S-lox-mCherry-t35S-lox-GFP-tAct (EC71194) the full sequences of the synthetic DNA inserted into pAGM4723 is shown below, with each component color-coded as follows:

nos promoter, nptII, ocs terminator, HSP terminator, CRE-U5intron-CRE, HS18.2 promoter, Act2 terminator, GFP, loxP, t35S, HDEL, 35S promoter, RC12A, minimal 35S promoter and mCherry.

tgccgaattcggatccggagcggagaattaagggagtcacgttatgacccccgccgatgacgcgggacaagccgttttacgtttggaactgacagaaccgcaacgttgaaggagccactgagccgcgggtttctggagtttaatgagctaagcacatacgtcagaaaccattattgcgcgttcaaaagtcgcctaaggtcactatcagctagcaaatatttcttgtcaaaaatgctccactgacgttccataaattcccctcggtatccaattagagtctcatattcactctcctatttttacaacaattaccaacaacaacaaacaacaaacaacattacaattacatttacaattaccatggttgaacaagatggattgcacgcaggttctccggccgcttgggtggagaggctattcggctatgactgggcacaacagacaatcggctgctctgatgccgccgtgttccggctgtcagcgcaggggcgcccggttctttttgtcaagaccgacctgtccggtgccctgaatgaactgcaggacgaggcagcgcggctatcgtggctggccacgacgggcgttccttgcgcagctgtgctcgacgttgtcactgaagcgggaagggactggctgctattgggcgaagtgccggggcaggatctcctgtcatctcaccttgctcctgccgagaaagtatccatcatggctgatgcaatgcggcggctgcatacgcttgatccggctacctgcccattcgaccaccaagcgaaacatcgcatcgagcgagcacgtactcggatggaagccggtcttgtcgatcaggatgatctggacgaagagcatcaggggctcgcgccagccgaactgttcgccaggctcaaggcgcgcatgcccgacggcgaggatctcgtcgtgactcatggcgatgcctgcttgccgaatatcatggtggaaaatggccgcttttctggattcatcgactgtggccggctgggtgtggcggaccgctatcaggacatagcgttggctacccgtgatattgctgaagagcttggcggcgaatgggctgaccgcttcctcgtgctttacggtatcgccgctcccgattcgcagcgcatcgccttctatcgccttcttgacgagttcttctgagcgggactctggggttcgctagagtcctgctttaatgagatatgcgagacgcctatgatcgcatgatatttgctttcaattctgttgtgcacgttgtaaaaaacctgagcatgtgtagctcagatccttaccgccggtttcggttcattctaatgaatatatcacccgttactatcgtatttttatgaataatattctccgttcaatttactgattgtaccctactacttatatgtacaatattaaaatgaaaacaatatattgtgctgaataggtttatagcgacatctatgatagagcgccacaataacaaacaattgcgttttattattacaaatccaattttaaaaaaagcggcagaaccggtcaaacctaaaagactgattacataaatcttattcaaatttcaaaagtgccccaggggctagtatctacgacacaccgagcggcgaactaataacgctcactgaagggaactccggttccccgccggcgcgcatgggtgagattccttgaagttgagtattggccgtccgctctaccgaaagttacgggcaccattcaacccggtccagcacggcggccgggtaaccgacttgctgccccgagaattatgcagcatttttttggtgtatgtgggccccaaatgaagtgcaggtcaaaccttgacagtgacgacaaatcgttgggcgggtccagggcgaattttgcgacaacatgtcgaggctcagccgctgcaagaattcaagcttagcgcttatctttaatcatattccatagtccataccatagcacatacagtagttatatgctgcagaagagatccaacaaaacattcacaatggattatagaaacatttgtttattcattataatgagatcttacattcatttaatattagaaaaagccacaaattcataacacaacaagccaagaaaaaaacacaaacttaagcacacaagctttttatttgacacaccaaatatttcatcttcatcttcatataagcctaatcgccatcttccagcaggcgcaccattgcccctgtttcactatccaggttacggatatagttcatgacaatatttacattggtccagccaccagcttgcatgatctccggtattgaaactccagcgcgggccatatctcgcgcggctccgacacgggcactgtgtccagaccaggccaggtatctctgaccagagtcatccttagcgccgtaaatcaatcgatgagttgcttcaaaaatcccttccagggcgcgagttgatagctggctggtggcagatggcgcggcaacaccattttttctgacccggcaaaacaggtagttattcggatcatcagctacaccagaaacggaaatccatcgctcgaccagtttagttacccccaggctaagtgccttctctacacctgcggtgctaaccagcgttttcgttctgccaatatggattaacattctcccaccgtcagtacgtgagatatctttaaccctgatcctggcaatttcggctatacgtaacagggtgttataagcaatccccagaaatgccagattacgtatatcctggcagcgatcgctattttccatgagtgaacgaacctggtcgaaatcagtgcgttcgaacgctagagcctgttttgcacgttcaccggcatcaacgttttcttttcggatccgccgcataaccagtgaaacagcattgctgtcacttggtcgtggcagcccggaccgacgatgaagcatgtttagctggcccaaatgttgctggatagtttttactgccagaccgcgcgcctgcacatcaacaaattttggtcatatattagaaaagttataaattaaaatatacacacttataaactacagaaaagcaattgctatatactacattcttttattttgaaaaaaatatttgaaatattatattactactaattaatgataattattatatatatatcaaaggtagaagcagaaacttacctgaagatatagaagataatcgcgaacatcttcaggttctgcgggaaaccatttccggttattcaacttgcaccatgccgcccacgaccggcaaacggacagaagcattttccaggtatgctcagaaaacgcctggcgatccctgaacatgtccatcaggttcttgcgaacctcatcactcgttgcatcgaccggtaatgcaggcaaattttggtgtacggtcagtaaattggacattggttcgttgcttttcgggagacttttggttcttgattttcctgctgatttgatctgattagcaaaggacatatttatagggaagttagaggatgaagagagaatgttcttcgagtttcttgaatagaatagaaaatgctcctttttctaaaaccttcgcttggagtctttagaacacatacacaatgatcactgtggtgaaatgaccagatttttcttggcatttcaggaagtttcgtttgttgaaacaagaccgaaatgcaatccttggttacaattaaaactacgtgccgttttgctttgttgttgtattatcctcttcgagatacgggctcagtgaagcggatcttctttgcaaaagcccatttcttaataggtcggaagtggaatttaagaaaatctaactcagagaaaagaggacaaaatcaagatttaagattattagcttttcgccaaaggaaaactatagtaagttctcgcaataacagttggcactaaatttccgagaatgtcaacaatcttttaatggatttttttgtctccatatatacaagaactgagccacgtttttgtgacaaacatggacaaaatatttactaaacaagtaaactaaaaaaaaaaaaatctaaatgattcgactcaagtatcagttactgtgatcaaaatctcaacttatttattgcctgttcatgtcatgcttgaaccagaagaaatgacgggctccactagaattcgagctcagcgaaatggtgcgatcttaaaatatctgactttgtgctagtttaagttcatttgtgaatggaacacatgtaacgctttaactattcagaatatcagttccaagtgaagatggaggtagcaaaagtaagactaataaccaaatcaataaaaatcttatacaatacttatattaacattgcaaagagtttcaaggtttttcttctgagttttgaaagaaattatagggaacaaaaggaataaagaggcatcaattcgatcactcagagctacaaaacaatgggactaaaacgcaaaacgaaagcggtttttttttttgaaacgcagacgtaagtaaaaacccagagagtttgtcacacacaagtgcatcatagaaacgaaaacaaaaagggaaatgaaacaaacaaatggagaagcaaatataaaagaaaaaaagaaactttgatcccattcataaaaccccagctttttaagcctttgatcttgagagcaagcttaaagctcatcatggccacccttgtacagctcgtccatgccgagagtgatcccggcggcggtcacgaactccagcaggaccatgtgatcgcgcttctcgttggggtctttgctcagggcggactgggtgctcaggtagtggttgtcgggcagcagcacggggccgtcgccgatgggggtgttctgctggtagtggtcggcgagctgcacgctgccgtcctcgatgttgtggcggatcttgaagttcaccttgatgccgttcttctgcttgtcggccatgatatagacgttgtggctgttgtagttgtactccagcttgtgccccaggatgttgccgtcctccttgaagtcgatgcccttcagctcgatgcggttcaccagggtgtcgccctcgaacttcacctcggcgcgggtcttgtagttgccgtcgtccttgaagaagatggtgcgctcctggacgtagccttcgggcatggcggacttgaagaagtcgtgctgcttcatgtggtcggggtagcggctgaagcactgcacgccgtaggtcagggtggtcacgagggtgggccagggcacgggcagcttgccggtggtgcagatgaacttcagggtcagcttgccgtaggtggcatcgccctcgccctcgccggacacgctgaacttgtggccgtttacgtcgccgtccagctcgaccaggatgggcaccaccccggtgaacagctcctcgcccttgctcaccatacctccgaattcggccgaggataatgataggagaagtgaaaagatgagaaagagaaaaagattagttttcattcaacccttaatataacttcgtataatgtatgctatacgaagttattaggtcagcgatctggattttagtactggattttggttttaggaattagaaattttattgatagaagtattttacaaatacaaatacatactaagggtttcttatatgctcaacacatgagcgaaaccctataggaaccctaattcccttatctgggaactactcacacattattatggagaaactcgagcttgtcgatcgactctagctagagaagcttaaagctcatcatggccacccttgtacagctcgtccatgccgccggtggagtggcggccctcggcgcgttcgtactgttccacgatggtgtagtcctcgttgtgggaggtgatgtccaacttgatgttgacgttgtaggcgccgggcagctgcacgggcttcttggccttgtaggtggtcttgacctcagcgtcgtagtggccgccgtccttcagcttcagcctctgcttgatctcgcccttcagggcgccgtcctcggggtacatccgctcggaggaggcctcccagcccatggttttcttctgcattacggggccgtcggaggggaagttggtgccgcgcagcttcaccttgtagatgaactcgccgtcctgcagggaggagtcctgggtcacggtcaccacgccgccgtcctcgaagttcatcacgcgctcccacttgaagccctcggggaaggacagcttcaagtagtcggggatgtcggcggggtgcttcacgtaggccttggagccgtacatgaactgaggggacaggatgtcccaggcgaagggcagggggccacccttggtcaccttcagcttggcggtctgggtgccctcgtaggggcggccctcgccctcgccctcgatctcgaactcgtggccgttcacggagccctccatgtgcaccttgaagcgcatgaactccttgatgatggccatgttatcctcctcgcccttgctcacacctccgaattcggccgaggataatgataggagaagtgaaaagatgagaaagagaaaaagattagttttcattcaacccttaatataacttcgtataatgtatgctatacgaagttattaggtcagtagcgtgtcctctccaaatgaaatgaacttccttatatagaggaagggtcttgcgaaggatagtgggattgtgcgtcatcccttacgtcagtggagatgtcacatcaatccacttgctttgtagacgtggttggaacctcttctttttccacgatgctcctcgtgggtgggggtccatctttgggaccactgtcggcagagagatcttgaatgatagcctttcctttatcgcaatgatggcatttgtaggagccaccttccttttctactgtcctttcgatgaagtgacagatagctgggcaatggaatccgaggaggtttcccgaaattatcctttgttgaaaagtctcaatagccctttgatcttctgagactgtatctttgacatttttggagtagaccagagtgtcgtgctccaccatgttgacctcc
