# Supplementary material for: Shaping of a three-dimensional carnivorous trap through modulation of a planar growth mechanism
Source: PLoS Biol. 2019 Oct 10;17(10):e3000427. doi: 10.1371/journal.pbio.3000427 (PMC6786542; doi:10.1371/journal.pbio.3000427)
Supplement: S2 Methods — (DOCX) [file pbio.3000427.s052.docx]

## S2 Methods. Full sequence for EC71257

To produce the pL2B-KAN-p35S-GFP-RC12A-t35S-DR5-mCherry-t35S (EC71257) the full sequences of the synthetic DNA inserted into pAGM4723 is shown below, with each component color-coded as follows:

nos promoter, nptII, ocs terminator, GFP, t35S, 35S promoter, RC12A, minimal 35S promoter, dr5 Promoter and mCherry.

ggagcggagaattaagggagtcacgttatgacccccgccgatgacgcgggacaagccgttttacgtttggaactgacagaaccgcaacgttgaaggagccactgagccgcgggtttctggagtttaatgagctaagcacatacgtcagaaaccattattgcgcgttcaaaagtcgcctaaggtcactatcagctagcaaatatttcttgtcaaaaatgctccactgacgttccataaattcccctcggtatccaattagagtctcatattcactctcctatttttacaacaattaccaacaacaacaaacaacaaacaacattacaattacatttacaattaccatggttgaacaagatggattgcacgcaggttctccggccgcttgggtggagaggctattcggctatgactgggcacaacagacaatcggctgctctgatgccgccgtgttccggctgtcagcgcaggggcgcccggttctttttgtcaagaccgacctgtccggtgccctgaatgaactgcaggacgaggcagcgcggctatcgtggctggccacgacgggcgttccttgcgcagctgtgctcgacgttgtcactgaagcgggaagggactggctgctattgggcgaagtgccggggcaggatctcctgtcatctcaccttgctcctgccgagaaagtatccatcatggctgatgcaatgcggcggctgcatacgcttgatccggctacctgcccattcgaccaccaagcgaaacatcgcatcgagcgagcacgtactcggatggaagccggtcttgtcgatcaggatgatctggacgaagagcatcaggggctcgcgccagccgaactgttcgccaggctcaaggcgcgcatgcccgacggcgaggatctcgtcgtgactcatggcgatgcctgcttgccgaatatcatggtggaaaatggccgcttttctggattcatcgactgtggccggctgggtgtggcggaccgctatcaggacatagcgttggctacccgtgatattgctgaagagcttggcggcgaatgggctgaccgcttcctcgtgctttacggtatcgccgctcccgattcgcagcgcatcgccttctatcgccttcttgacgagttcttctgagcgggactctggggttcgctagagtcctgctttaatgagatatgcgagacgcctatgatcgcatgatatttgctttcaattctgttgtgcacgttgtaaaaaacctgagcatgtgtagctcagatccttaccgccggtttcggttcattctaatgaatatatcacccgttactatcgtatttttatgaataatattctccgttcaatttactgattgtaccctactacttatatgtacaatattaaaatgaaaacaatatattgtgctgaataggtttatagcgacatctatgatagagcgccacaataacaaacaattgcgttttattattacaaatccaattttaaaaaaagcggcagaaccggtcaaacctaaaagactgattacataaatcttattcaaatttcaaaagtgccccaggggctagtatctacgacacaccgagcggcgaactaataacgctcactgaagggaactccggttccccgccggcgcgcatgggtgagattccttgaagttgagtattggccgtccgctctaccgaaagttacgggcaccattcaacccggtccagcacggcggccgggtaaccgacttgctgccccgagaattatgcagcatttttttggtgtatgtgggccccaaatgaagtgcaggtcaaaccttgacagtgacgacaaatcgttgggcgggtccagggcgaattttgcgacaacatgtcgaggctcagccgctgcaagaattcaagcttagcgatctggattttagtactggattttggttttaggaattagaaattttattgatagaagtattttacaaatacaaatacatactaagggtttcttatatgctcaacacatgagcgaaaccctataggaaccctaattcccttatctgggaactactcacacattattatggagaaactcgagcttgtcgatcgactctagctagagaagcaatggttaatggtggtcctgtgatccaccaaggcaacacaatttccaatttattgtaataaaccaactatatttttaaaaaatcttcacacaaagtacaaaccaacgctctcttcttttatatcaacacaacatttatataacaatgcaaacacaacagtgatgattaattaaattacttgaaacataaaaactctgatgaaagaaaaagaaacgctgaatcagtcacaaagataggagaacacgacggaacagctgttcctgcacaataaacaacaaatgtaagtaaagttaaaaagtttggtcattaactagatatttatctctatccacattagagtcttattactctcttttgtgtcagaaatttctcataatcttatcttattaataagtagatatttatctctatctgtacacattaattatgtggtttgaacaatatcttaatttattatcataataatatttataacttgaaaccatgttttcatagcataattttcgtttgctttaaaccaaattatccgaattaacatgatctgaaatgtgtaggtttaccaaacatttacttaaactttcccaatgaaaaagcttcatacaagaaaactatatcaaaaatgactctgagagtgagccaaagggtaaagagagagagagagagagagagcttacaaggagatgatgatagatggtaaatcatttggtgaggacataaatggcgtatatgatcccaggaatatacccaagtagcgtcaaaaccaaacatatccaaaactcaacctgcaacaaaattcataatttcaacttttaatttctagtgtggataaatgtaaaaaaagaaccatatttgactaaagtataagattaattaaccaaaataaaactcagtctaaatatgagaggaaagataaataccccgcaaccaaatctgagaaagacaccgagaggaggcaagaggatggcgataataatatcaacgaaagtagctgtactcatggatccggccgctgccgcagcggcagccgcagctgctccggaacctcccttgtacagctcgtccatgccgagagtgatcccggcggcggtcacgaactccagcaggaccatgtgatcgcgcttctcgttggggtctttgctcagggcggactgggtgctcaggtagtggttgtcgggcagcagcacggggccgtcgccgatgggggtgttctgctggtagtggtcggcgagctgcacgctgccgtcctcgatgttgtggcggatcttgaagttcaccttgatgccgttcttctgcttgtcggccatgatatagacgttgtggctgttgtagttgtactccagcttgtgccccaggatgttgccgtcctccttgaagtcgatgcccttcagctcgatgcggttcaccagggtgtcgccctcgaacttcacctcggcgcgggtcttgtagttgccgtcgtccttgaagaagatggtgcgctcctggacgtagccttcgggcatggcggacttgaagaagtcgtgctgcttcatgtggtcggggtagcggctgaagcactgcacgccgtaggtcagggtggtcacgagggtgggccagggcacgggcagcttgccggtggtgcagatgaacttcagggtcagcttgccgtaggtggcatcgccctcgccctcgccggacacgctgaacttgtggccgtttacgtcgccgtccagctcgaccaggatgggcaccaccccggtgaacagctcctcgcccttgctcaccattgcgtgtcctctccaaatgaaatgaacttccttatatagaggaagggtcttgcgaaggatagtgggattgtgcgtcatcccttacgtcagtggagatgtcacatcaatccacttgctttgtagacgtggttggaacctcttctttttccacgatgctcctcgtgggtgggggtccatctttgggaccactgtcggcagagagatcttgaatgatagcctttcctttatcgcaatgatggcatttgtaggagccaccttccttttctactgtcctttcgatgaagtgacagatagctgggcaatggaatccgaggaggtttcccgaaattatcctttgttgaaaagtctcaatagccctttgatcttctgagactgtatctttgacatttttggagtagaccagagtgtcgtgctccaccatgttgacctccactagaattcgagctcagcgatctggattttagtactggattttggttttaggaattagaaattttattgatagaagtattttacaaatacaaatacatactaagggtttcttatatgctcaacacatgagcgaaaccctataggaaccctaattcccttatctgggaactactcacacattattatggagaaactcgagcttgtcgatcgactctagctagagaagcttacttgtacagctcgtccatgccgccggtggagtggcggccctcggcgcgttcgtactgttccacgatggtgtagtcctcgttgtgggaggtgatgtccaacttgatgttgacgttgtaggcgccgggcagctgcacgggcttcttggccttgtaggtggtcttgacctcagcgtcgtagtggccgccgtccttcagcttcagcctctgcttgatctcgcccttcagggcgccgtcctcggggtacatccgctcggaggaggcctcccagcccatggttttcttctgcattacggggccgtcggaggggaagttggtgccgcgcagcttcaccttgtagatgaactcgccgtcctgcagggaggagtcctgggtcacggtcaccacgccgccgtcctcgaagttcatcacgcgctcccacttgaagccctcggggaaggacagcttcaagtagtcggggatgtcggcggggtgcttcacgtaggccttggagccgtacatgaactgaggggacaggatgtcccaggcgaagggcagggggccacccttggtcaccttcagcttggcggtctgggtgccctcgtaggggcggccctcgccctcgccctcgatctcgaactcgtggccgttcacggagccctccatgtgcaccttgaagcgcatgaactccttgatgatggccatgttatcctcctcgcccttgctcaccattcctctccaaatgaaatgaacttccttatatagaggaagggtcttgcggggctgcaggaattaattcgatatcaagcttatcgaggcctgcccccttttgtctcccttttgtctcccttttgtctcccttttgtctcccttttgtctcccttttgtctcccttttgtctcccttttgtctcccttttgtctccctgggctgcgataccgtcgacctccttacgaggatgcacatgtgaccgaggga
