# Supplementary material for: Shaping of a three-dimensional carnivorous trap through modulation of a planar growth mechanism
Source: PLoS Biol. 2019 Oct 10;17(10):e3000427. doi: 10.1371/journal.pbio.3000427 (PMC6786542; doi:10.1371/journal.pbio.3000427)
Supplement: S1 Resources — (DOCX) [file pbio.3000427.s053.docx]

| **Key Resources table** |  |  |
| --- | --- | --- |
| **Reagent or resource** | **Source** | **Identifier** |
| **Experimental Models: Organisms/Strains** |  |  |
| Utricularia gibba Bergh Apton (BA) accession | The Fly Trap Plants, Cookes Road, Bergh Apton, NR15 1BA, UK | SM7 |
| pL2B-KAN-Cre-p35S-lox-mCherry-t35S-lox-GFP-tAct | This paper | EC71194 |
| pL2B-KAN-p35S-GFP-RC12A-t35S-DR5-mCherry-t35S | This paper | EC71257 |
| **Software and Algorithms** |  |  |
| VolViewer | Lee et al, 2006 | [http://cmpdartsvr3.cmp.uea.ac.uk/wiki/BanghamLab/index.php/VolViewer#Download](http://cmpdartsvr3.cmp.uea.ac.uk/wiki/BanghamLab/index.php/Software#Viewing_and_measuring_volume_images:_VolViewer) |
| VolViewer Arrows | This paper | <https://doi.org/10.6084/m9.figshare.8966153.v1> Fig12_S8.7z |
| Quadrifid script 6 | This paper | <https://doi.org/10.6084/m9.figshare.8966153.v1> Fig12_S8.7z |
| Segmentation scripts |  | <https://github.com/jfozard/gibba_analysis> |
| Growing Polarised Tissue (GPT) Framework | Kennaway et al, 2011 | <http://cmpdartsvr3.cmp.uea.ac.uk/wiki/BanghamLab/index.php/Software> |
| **Data** |  |  |
| Segmentation results | This paper | <https://doi.org/10.6084/m9.figshare.8966153.v1> Fig9_10_S4_S6.7z |
| Models | This paper | <http://cmpdartsvr3.cmp.uea.ac.uk/wiki/BanghamLab/index.php/Software#GFtbox%7CPublished>  and <https://doi.org/10.6084/m9.figshare.8966153.v1> Models.7z |
| Confocal data | This paper | <https://doi.org/10.6084/m9.figshare.8966153.v1> Additional Data.7z |
| OPT data | This paper | <https://doi.org/10.6084/m9.figshare.8966153.v1> Additional Data.7z |
| **Chemicals** |  |  |
| Propidium Iodide | Merck | Cat# P4170 |
| Ethephon | Merck | Cat# C0143 |
| **Critical Commercial Assay** |  |  |
| iDNA genetics copy number analysis | iDNA genetics | N/A |
